# Absolute Configuration of the Invasive Mealybug *Delottococcus aberiae* (De Lotto) Sex Pheromone: Synthesis and Bioassay of Both Enantiomers

**DOI:** 10.1021/acs.jafc.4c05469

**Published:** 2024-09-19

**Authors:** Javier Marzo Bargues, Sandra Vacas, Ismael Navarro Fuertes, Jaime Primo, Antonio Abad-Somovilla, Vicente Navarro-Llopis

**Affiliations:** † Ecología y Protección Agrícola SL, Pol. Ind. Ciutat de Carlet, 46240 Carlet, Valencia, Spain; ‡ Departamento de Química Orgánica, Universitat de València, Dr Moliner 50, 46100 Burjassot, Valencia, Spain; § CEQA-Instituto Agroforestal del Mediterráneo, Universitat Politècnica de València, Camino de Vera s/n, edificio 6C-5a̲ planta, 46022 Valencia, Valencia, Spain

**Keywords:** D. aberiae, pheromone, insect attractant, enantiomers, diastereomeric resolution, necrodol skeleton

## Abstract

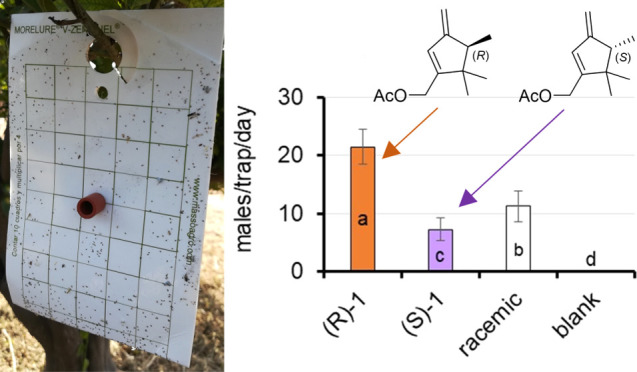

The mealybug *Delottococcus aberiae* (De Lotto) (Hemiptera: Pseudococcidae) is an invasive pest reported in Europe at the end of the first decade of the 2000s, causing severe damage to citrus production in eastern Spain. In a previous work, (4,5,5-trimethyl-3-methylenecyclopent-1-en-1-yl)methyl acetate was identified as the sex pheromone emitted by females, a new compound with an unusual β-necrodol skeleton possessing one stereocenter. This compound was assigned to the (−)-enantiomer but the absolute configuration was then not reported. In the present study, enantiomeric pure samples of both enantiomers were synthesized. X-ray diffraction analysis allowed the (−)-enantiomer, identical to the one emitted by virgin *D. aberiae* females, to be unequivocally identified as (−)-(*R*)-(4,5,5-trimethyl-3-methylenecyclopent-1-en-1-yl)methyl acetate. Bioassays carried out to test the activity of both enantiomers under field conditions suggest that the presence of the (+)-(*S*)-enantiomer has detrimental effects on the activity of the racemates.

## Introduction

*Delottococcus aberiae* (De Lotto) (Hemiptera: Pseudococcidae) is a polyphagous pest native to sub-Saharan Africa.^1^ It was first detected in Europe, specifically in Spain, at the end of the first decade of 2000.^2^*D. aberiae* has been found on various tropical and subtropical crops, including coffee, guava, pear, and olive.^3^ It has also been recorded in more than 25 different botanical families.^4^ However, it was upon its arrival in the Mediterranean basin, with the growing restrictions on the use of pesticides imposed by European authorities, the lack of specific management tools and effective natural enemies^5^ among other causes, that citrus crops were significantly affected. *D. aberiae* feeds on the sap of fruits, causing deformation and/or reduction in their size. All types of citrus are susceptible to its attack, but the symptoms vary depending on the variety. Controlling this pest is challenging due to its cryptic habits, reproductive capacity, and the lack of effective pesticides. Interestingly, the sexual reproduction of this pseudococcid and the great ability of its sex pheromone to attract males,^6,7^ provide a highly specific and effective tool for detecting, monitoring and potentially controlling this pest.

**Figure 1:**
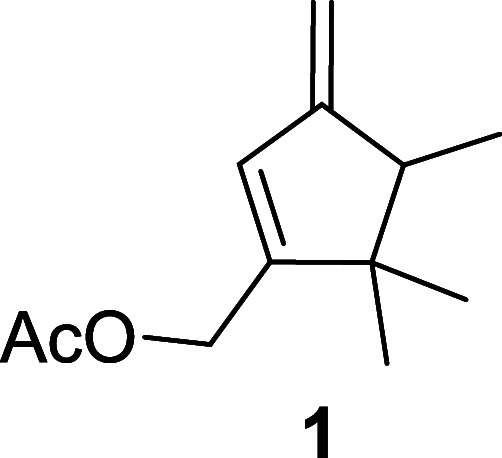
*D. aberiae* sex pheromone.

The chemical structure of the sex pheromone of *D. aberiae* was elucidated by our group as (4,5,5-trimethyl-3-methylenecyclopent-1-en-1-yl)methyl acetate **1** (Figure 1),^7^ an irregular monoterpenoid with an unusual β-necrodol skeleton. The first members of this class of monoterpenes, α- and β-necrodols, were discovered by Eisner and Meinwald in the defensive spray of the red-line carrion beetle, *Necrodes surinamensis* (Fabricius) (Coleoptera: Silphidae).^8^ Later, two more examples of sex pheromones belonging to mealybug species were described: *trans*-α-necrodyl isobutyrate from *Pseudococcus maritimus* (Ehrhorn) (Hemiptera: Pseudococcidae)^9^ and γ-necrodol and γ-necrodyl isobutyrate from *Nipaecoccus viridis* (Newstead).^10^ Necrodane-type monoterpenes have been also found into the plant kingdom, with examples such as *trans*-α-necrodol in *Lavandula stoechas* subsp. *luisieri* (Rozeira) (Lamiales: Lamiaceae)^11^ and *cis*-α-necrodol in *Evolvulus alsinoides* (L.) (Solanales: Convolvulaceae),^12^ although their presence in essential oils is not common.

In a previous work by our group, a confirmatory synthesis of the sex pheromone of *D. aberiae* as a racemic mixture was described, together with its activity in both field and laboratory tests (Figure 1).^7^ The specific rotation of the emitted sex pheromone was assigned to the (−)-levorotatory series by comparison with a natural sample using chiral gas and liquid chromatography techniques. Unfortunately, due to the limited quantity obtained for each enantiomer, neither the absolute configuration nor the enantiomeric purity was determined with certainty. Understanding the relationship between chirality and bioactivity of sex pheromones is essential when they are used as tools in pest management. This is particularly important when male attraction is required for the success of these management techniques (mass trapping or attract and kill), because the presence of the non-natural enantiomer in the sex pheromone formulations sometimes reduce, enhance, restore, or not influence the effectiveness of a trap or a control device.^13^ Therefore, the determination of the absolute configuration of *D. aberiae’s* pheromone, as well as testing the attractant activity of each enantiomer, provide useful information to gain knowledge about the behavior of the pest and to develop pest management strategies for this mealybug.

## Materials and Methods

### Chemicals

All reagents were purchased from Merck (Madrid, Spain) and used without further purification. All organic solvents required in anhydrous conditions were dried and distilled before use. Toluene and tetrahydrofuran (THF) were distilled over sodium and benzophenone under a nitrogen atmosphere just before use. Dichloromethane (CH_2_Cl_2_) was distilled using calcium hydride (CaH_2_) in the same manner. Operations involving air- or moisture-sensitive reagents were conducted under an inert atmosphere of dry nitrogen, utilizing syringes and oven-dried glassware (heated to 130 °C). Reactions were monitored by thin-layer chromatography on precoated silica plates (0.25 mm layer thickness, Silica Gel 60 F_254_) with ultraviolet (UV) light as the visualizing agent. Developing agents included ethanolic phosphomolybdic acid or aqueous ceric ammonium molybdate solutions, along with heat. A Büchi Rotavapor R-100 was utilized to distill the organic phases obtained after reaction work up, with a fixed vacuum of 80 mbar and operating temperatures between 30 and 40 °C. Crude products were purified by column flash chromatography using silica gel Merck 9385 (230–400 mesh).

### Instrumental Analysis

Melting points (Mp) were determined using a Büchi M-560 apparatus and remain uncorrected. Optical rotations were recorded on a PerkinElmer Mod. 343 polarimeter at a temperature of 20 °C, using a 1 dm cell and the specified solvent in each case. Concentrations of the solutions are expressed in g/100 mL. ^1^H/^13^C NMR spectra were recorded at 298 K in the indicated solvent, at 300/75 MHz (Bruker Avance DPX300 spectrometer) or 500/125 MHz (Bruker Avance DRX500). Chemical shifts are expressed in ppm (δ scale) relative to the residual solvent, which serves as the internal reference in all cases (7.27/77.00 ppm for the ^1^H/^13^C spectra in CDCl_3_). Carbon substitution degrees were determined using DEPT pulse sequences. High-resolution mass spectra (HRMS) were obtained via electrospray ionization (ESI) mode using a premier quadrupole time of flight (Q-TOF) mass spectrometer equipped with an electrospray source (Waters, Manchester, U.K.). The obtained data are expressed as mass-to-charge ratio (*m*/*z*). Enantiomeric excess of synthetic samples, as well as levo (−) and dextro (+) enantiomers assignation, was determined by their injection into an InertCap CHIRAMIX chiral capillary column (30 m × 0.25 mm i.d. × 0.25 μm; GL Sciences Inc., Tokyo, Japan) installed in a Clarus 590 GC instrument (PerkinElmer Inc., Wellesley, PA) equipped with a flame ionization detector (FID) and a programmable split/splitless built-in injector, both set at 250 °C. The GC oven temperature was raised at 0.6 °C/min from 50 to 115 °C and then at 25 °C/min to 150 °C, which was finally held for 10 min. Carrier gas was helium at 1 mL/min flow rate. X-ray diffraction analysis was performed using a double source single crystal diffraction (Mo/Cu) model SupernovaDual (Agilent, Rigaku).

### Synthetic Route

**Figure 2:**
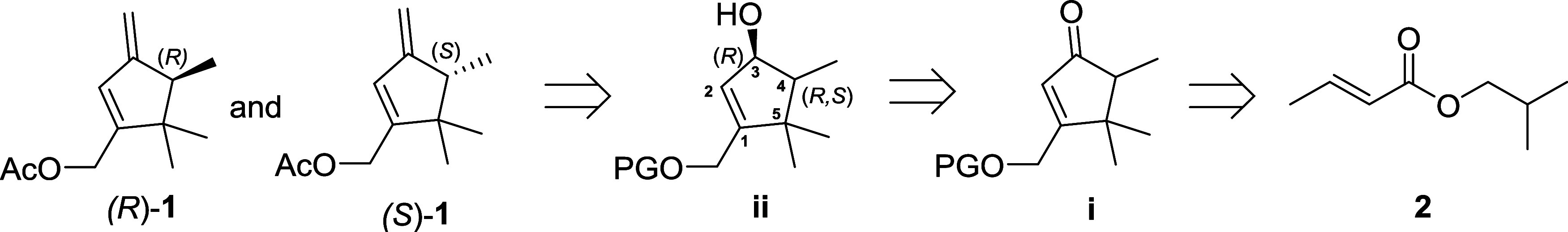
Retrosynthetic strategy for the preparation of enantiomeric (*R*)-**1** and (*S*)-**1** (PG: hydroxyl protecting group).

**Figure 3:**
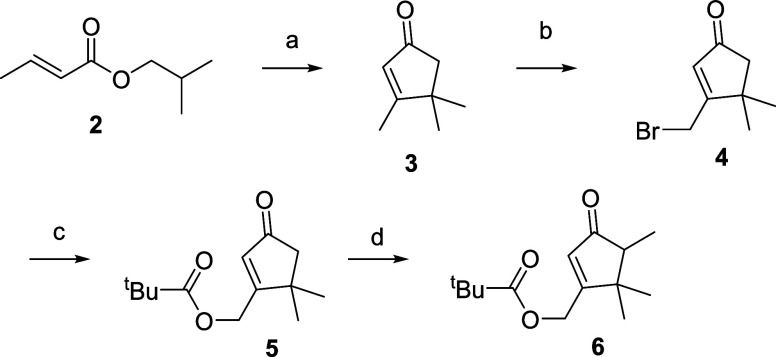
Synthesis of cyclopentenone **6** from isobutyl (*E*)-but-2-enoate (**2**). Reagents and conditions: (a) polyphosphoric acid, 95 °C, 2h, 55%; (b) NBS, CH_2_Cl_2_, *h*ν, rt, 4h, 60%; (c) potassium pivalate, TBAB, MTBE, rt, 8h, 85%; (d) lithium bis(trimethylsilyl)amide, MeI, THF, −35 °C, 1 h, 62%.

**Figure 4:**
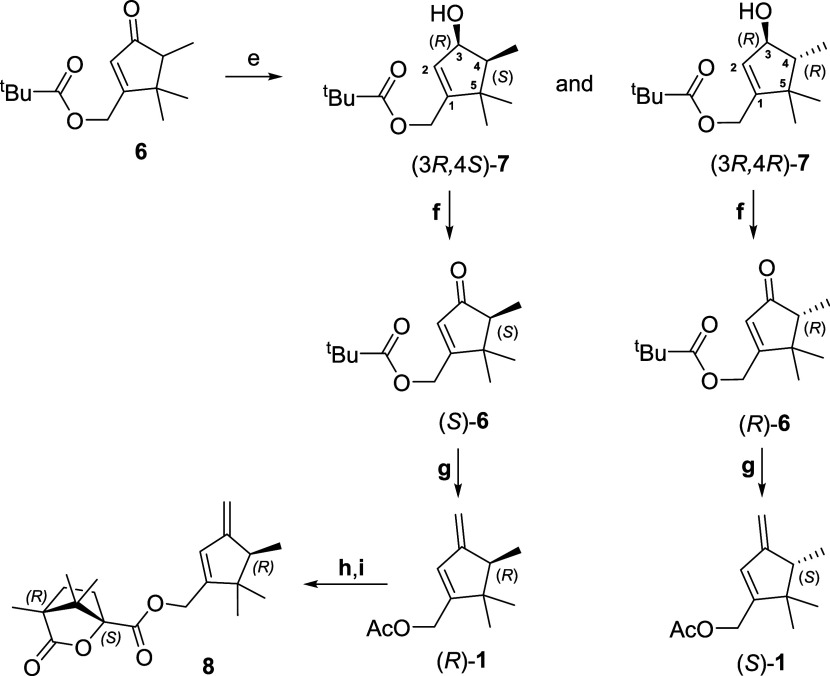
Completion of the synthesis of both enantiomers of the sex pheromone of *D. aberiae* from cyclopentenone **6** and preparation of camphonate ester **8**. Reagents and conditions: (e) BH_3_·SMe_2_, (*S*)-(−)-CBS-oxazaborolidine, THF, −40 °C, 1 h, 86%; (f) PCC, CH_2_Cl_2_, rt, 1 h, 88%; (g) MeMgCl, Cp_2_TiCl_2_, 90 °C, 4 h, then MeMgCl, EtOAc, rt, 18 h, 66%; (h) KOH, MeOH, rt, 45 min; (i) (1*S*)-(−)-camphanic chloride, DMAP, Et_3_N, CH_2_Cl_2_, rt, 5 h, 85%.

Sex pheromone **1** has been synthesized following the sequence set out in Figures 2, 3, and 4 (see Results and Discussion section) following the experimental procedures described below.

#### 3,4,4-Trimethylcyclopent-2-en-1-one (**3**)

Isobutyl crotonate (**2**; 10 g, 70 mmol) was slowly added to polyphosphoric acid (50 g) at 95 °C for 2 h. After continuous stirring at this temperature for 2 h, the solution was cooled and poured into water (70 mL) with stirring. The mixture was extracted with diethyl ether (2 × 30 mL), and the organic layers were successively washed with saturated aqueous NaHCO_3_ (20 mL) and brine (20 mL), dried over MgSO_4_, and concentrated under reduced pressure. The crude material was distilled under reduced pressure to give 6 g (55% yield) of **3** as a yellow oil. Its spectroscopic data were fully coincident with those previously described in the literature.^14^

#### 3-(Bromomethyl)-4,4-dimethylcyclopent-2-en-1-one (**4**)

A 100 mL Pyrex round-bottom flask containing a solution of **3** (1.00 g, 8 mmol) and NBS (1.85 g, 10.4 mmol, 1.3 equiv) in anhydrous CH_2_Cl_2_ (50 mL) was placed inside an irradiation chamber equipped with a 400 W visible lamp (HPI Plus, Koninklijke Philips NV, Amsterdam, The Netherlands). The flask was irradiated with stirring for 4 h at room temperature. After this period, the solution was poured into hexane (50 mL) and filtered. The organic solution was concentrated under vacuum, and the crude residue (ca. 1.70 g) was purified by flash column chromatography on silica gel, using a 9:1 mixture of hexane and Et_2_O as eluent, to give 970 mg (60% yield) of **4** as a yellow oil. Its spectroscopic data were fully coincident with those previously described in the literature.^7^

#### (5,5-Dimethyl-3-oxocyclopent-1-en-1-yl)methyl Pivalate (**5**)

Potassium pivalate (895 mg, 6.4 mmol, 1.3 equiv) was added to a solution of **4** (1.00 g, 4.9 mmol) and TBAB (16 mg, 0.05 mmol, 0.01 equiv) in MTBE (15 mL). The suspension was stirred for 8 h at room temperature and then poured into water (20 mL). The mixture was extracted with EtOAc (2 × 15 mL), and the combined organic layers were successively washed with saturated NaHCO_3_ (20 mL) and brine (20 mL), dried over MgSO_4_, and concentrated under reduced pressure. The crude residue obtained was purified by flash column chromatography on silica gel, using an 8:2 mixture of hexane and Et_2_O as eluent, to give 935 mg (85% yield) of **5** as a yellow oil. ^1^H NMR (300 MHz, CDCl_3_) (Figure S1) δ 5.97 (1H, t, *J* = 1.7 Hz, H-2), 4.90 (2H, d, *J* = 1.7 Hz, CH_2_O), 2.36 (2H, s, H-4), 1.29 (6H, s, 2xMe-5), 1.24 (9H, s, *t*-Bu); ^13^C NMR (75 MHz, CDCl_3_) (Figure S2) δ 206.8 (C-3), 181.8 (CO_2_), 178.0 (C-1), 127.2 (C-2), 60.2 (CH_2_O), 52.2 (C-4), 41.9 (C-5), 39.0 (Me_3_*C*), 27.4 (2xMe-5), 27.3 (*Me*_3_C); HRMS (TOF MS ESI^+^) calcd for C_13_H_21_O_3_ [M + H]^•+^ 225.1485, found 225.1496.

#### (4,5,5-Trimethyl-3-oxocyclopent-1-en-1-yl)methyl Pivalate (**6**)

A solution of **5** (1.00 g, 4.5 mmol) in THF (2 mL) was added dropwise to a solution of lithium bis(trimethylsilyl)amide solution (20 mL, 0.5 M, 10 mmol) at −35 °C. After 40 min, the solution was warmed to −30 °C, and MeI (13.5 mmol, 0.85 mL, 3 equiv) was added. The mixture was kept at this temperature for 1 h and then gradually warmed to −15 °C. The reaction was quenched with saturated aqueous NH_4_Cl (5 mL), poured into water (15 mL), and extracted with EtOAc (2 × 20 mL). The combined organic layers were successively washed with aqueous 1 M HCl (20 mL), aqueous saturated NaHCO_3_ (20 mL), and brine (20 mL), dried over MgSO_4_, and concentrated under reduced pressure. The crude residue was purified by flash column chromatography on silica gel, using an 8:2 mixture of hexane and Et_2_O as eluent, to give 665 mg (62% yield) of **6** as a yellow oil. ^1^H NMR (300 MHz, CDCl_3_) (Figure S3) δ 5.99 (1H, t, *J* = 1.6 Hz, H-2), 4.92 (2H, dd, *J* = 3.8, 1.6 Hz, CH_2_O), 2.24 (1H, q, *J* = 7.4 Hz, H-4), 1.26 (3H, s, Me-5), 1.25 (9H, s, *t*-Bu), 1.11 (3H, s, Me’-5), 1.08 (3H, d, *J* = 7.5 Hz, Me-4). ^13^C NMR (75 MHz, CDCl_3_) (Figure S4) δ 209.1 (C-3), 180.4 (CO_2_), 178.0 (C-1), 125.9 (C-2), 60.57 (CH_2_O), 53.7 (C-4), 44.9 (C-5), 39.0 (Me_3_*C*), 27.3 (*Me*_3_C), 26.2 (Me-5), 23.9 (Me’-5), 9.7 (Me-4); HRMS (TOF MS ESI+) calcd for C_14_H_23_O_3_ [M + H]^•+^ 239.1642, found 239.1650.

#### ((3*R*,4*S*)-3-Hydroxy-4,5,5-trimethylcyclopent-1-en-1-yl)methyl Pivalate [(3*R*,4*S*)-**7**] and ((3*R*,4*R*)-3-Hydroxy-4,5,5-trimethylcyclopent-1-en-1-yl)methyl Pivalate [(3*R*,4*R*)-**7**]

A 2 M solution of the BH_3_·SMe_2_ complex in THF (1.05 mL, 2.1 mmol, 1 equiv) was added to a solution of (*S*)-(−)-2-methyl-CBS-oxazaborolidine (0.52 mmol, 0.25 equiv) in THF (30 mL) at −40 °C. Next, a solution of ketone **6** (500 mg, 2.1 mmol) in THF (1 mL) was added dropwise and stirred for 1 h. The reaction was quenched with MeOH (1 mL), poured into water (20 mL), and extracted with EtOAc (2 × 20 mL). The combined organic layers were washed with aqueous 1 M HCl (10 mL) and brine (20 mL). After drying over MgSO_4_, the solvent was eliminated under reduced pressure to afford a ca. 1:1 mixture of diastereomeric alcohols (3*R*,4S)-**7** and (3*R*,4*R*)-**7**; retention factor of 0.45 and 0.30 in thin layer chromatography, respectively, using silica gel plates and a 7:3 mixture of hexane and AcOEt as eluent. See Supporting Information for enantiomeric excess determination for both diastereomers, (Figure S18), which were separated by flash column chromatography on silica gel in a single purification cycle, using a 7:3 mixture of hexane and Et_2_O as eluent, to give, in order of elution, 210 mg of diastereomeric alcohol (3*R*,4*S*)-**7** and 225 mg of the diastereomer (3*R*,4*R*)-**7** as yellow oils (86% combined yield).

Physical and spectroscopic data of diastereomer (3*R*,4*S*)-**7**: ^1^H NMR (300 MHz, CDCl_3_) (Figure S5) δ 5.78 (1H, dt, *J* = 2.9, 1.7 Hz, H-2), 4.69–4.62 (2H, m, CH_2_O), 4.46 (1H, d, *J* = 4.0 Hz, H-3), 1.96–1.79 (1H, m, H-4), 1.23 (9H, s, *t*-Bu), 1.02 (6H, s, 2xMe-5), 1.01 (3H, d, *J* = 7.8 Hz, Me-4); ^13^C NMR (75 MHz, CDCl_3_) (Figure S6) δ 178.4 (CO_2_), 153.5 (C-1), 126.7 (C-2), 76.7 (C-3), 61.1 (CH_2_O), 48.8 (C-4), 46.5 (C-5), 39.0 (Me_3_*C*), 27.4 (*Me*_3_C), 25.6 (Me-5), 25.0 (Me’-5), 8.4 (Me-4); HRMS (TOF MS ESI^+^) calcd for C_14_H_28_NO_3_ [M + NH_4_]^•+^ 258.2064, found 258.2067; [α]_D_ −38.7 (c 0.32, CHCl_3_).

Physical and spectroscopic data of diastereomer (3*R*,4*R*)-**7**: ^1^H NMR (300 MHz, CDCl_3_) (Figure S7) δ 5.62 (1H, q, *J* = 1.6 Hz, H-2), 4.64–4.59 (2H, m, CH_2_O), 4.40–4.25 (1H, m, H-3), 1.74–1.61 (1H, m, H-4), 1.22 (9H, s, *t*-Bu), 1.06 (3H, d, *J* = 7.8 Hz, Me-4), 1.06 (3H, s, Me-5β), 0.90 (3H, s, Me-5α); ^13^C NMR (75 MHz, CDCl_3_) (Figure S8) δ 178.4 (CO_2_), 149.6 (C-1), 128.6 (C-2), 81.8 (C-3), 60.9 (CH_2_O), 55.3 (C-4), 46.4 (Me_3_*C*), 39.0 (C-5), 27.4 (*Me*_3_C), 26.2 (Me-5β), 22.2 (Me-5α), 11.2 (Me-4); HRMS (TOF MS ESI^+^) calcd for C_14_H_28_NO_3_ [M + NH_4_]^•+^ 258.2064, found 258.2069; [α]_D_ −41.3 (c 0.15, CHCl_3_).

#### (*S*)-(4,5,5-Trimethyl-3-oxocyclopent-1-en-1-yl)methyl Pivalate [(S)-**6**] and (*R*)-(4,5,5-trimethyl-3-oxocyclopent-1-en-1-yl)methyl Pivalate [(R)-**6**]

Pyridinium chlorochromate (PCC, 246 mg, 1.14 mmol, 1.1 equiv) was portionwise added over 20 min to a solution of (3*R*,4*S*)-**7** (250 mg, 1.04 mmol) in CH_2_Cl_2_ (30 mL). The suspension was stirred for an additional 1 h and directly concentrated under reduced pressure. The crude residue obtained was purified by flash column chromatography on silica gel, using an 8:2 mixture of hexane and Et_2_O as eluent, to yield 220 mg (88% yield) of ketone (*S*)-**6** as a yellow oil. The ^1^H (Figure S9) and ^13^C NMR spectra were identical to that of the racemic ketone **6**. [α]_D_ 5.9 (c 1.18, CHCl_3_).

The enantiomeric methyl ketone (*R*)-**6** (205 mg, 82% yield) was obtained from alcohol (3*R*,4*R*)-**7** (250 mg, 1.04 mmol) following the same procedure described above for the transformation of (3*R*,4*S*)-**7** into (*S*)-**6**. Its ^1^H (Figure S10) and ^13^C NMR spectra were identical to that of the racemic ketone **6**. [α]_D_ −6.8 (c 0.91, CHCl_3_).

#### (*R*)-(4,5,5-Trimethyl-3-methylenecyclopent-1-en-1-yl)methyl Acetate [(*R*)-**1**] and (*S*)-(4,5,5-Trimethyl-3-methylenecyclopent-1-en-1-yl)methyl Acetate [(*S*)-**1**]

A methylmagnesium chloride 3 M solution in THF (1.4 mL, 4.2 mmol) was added to a suspension of bis(cyclopentadienyl)titanium(IV) dichloride (522 mg, 2.1 mmol) in toluene (15 mL) at 0 °C. After 20 min of stirring, the solution was gradually warmed to room temperature, and a solution of ketone (*S*)-**6** (250 mg, 1.05 mmol) in dry toluene (2 mL) was added. The solution was heated to 90 °C for 4 h and then cooled to 0 °C. Next, an additional amount of the methylmagnesium chloride solution (2 mL, 6 mmol) was added, and after 20 min of stirring, EtOAc (10 mL) was slowly added. The suspension was stirred at room temperature for 18 h, then quenched with water (5 mL), followed by the addition of aqueous 3 M HCl (5 mL). The mixture was then extracted with EtOAc (2 × 10 mL), and the combined organic layers were successively washed with aqueous saturated NaHCO_3_ (10 mL) and brine (10 mL) and dried over MgSO_4_. The residue obtained after evaporation of the solvent under reduced pressure was purified by flash column chromatography on silica gel, using a 95:5 mixture of hexane and Et_2_O as eluent, to yield 134 mg (66% yield) of diene (*R*)-**1** as a yellow oil. ^1^H NMR (300 MHz, CDCl_3_) (Figure S11) δ 6.06 (1H, s, H-2), 4.86 (1H, d, *J* = 2.7 Hz, CH’H-3), 4.75–4.70 (2H, m, CH_2_O), 4.67 (1H, d, *J* = 1.6 Hz, CHH’-3), 2.45 (1H, qt, *J* = 7.2, 2.6 Hz, H-4), 2.10 (3H, s, MeCO_2_), 1.09 (3H, s, Me-5α), 1.04 (3H, d, *J* = 7.1 Hz, Me-4), 0.89 (3H, s, Me-5β). ^13^C NMR (75 MHz, CDCl_3_) (Figure S12) δ 170.9 (CO_2_), 156.5 (C-3), 152.9 (C-1), 129.0 (C-2), 103.0 (=CH_2_), 61.1 (CH_2_O), 49.2 (C-4), 47.6 (C-5), 26.1 (Me-5α), 22.3 (Me-5β), 21.1 (*Me*CO_2)_, 12.4. HRMS (TOF MS ESI^+^) calcd for C_12_H_19_O_2_ [M + H]^•+^ 195.1380, found 195.1375; [α]_D_ −40.0 (c 0.10, C_6_H_5_CH_3_).

The enantiomeric diene (*S*)-**1** (115 mg, 56% yield) was obtained from (*R*)-**6** (250 mg, 1.05 mmol) following a similar procedure to that described for the transformation of (*S*)-**6** into (*R*)-**1**. The NMR (Figure S13) and MS spectroscopic data of these enantiomers were completely coincident with those of (*R*)-**1**. [α]_D_ 41.2 (c 0.40, C_6_H_5_CH_3_).

#### ((*R*)-4,5,5-Trimethyl-3-methylenecyclopent-1-en-1-yl)methyl (1*S*,4*R*)-4,7,7-Trimethyl-3-oxo-2-oxabicyclo[2.2.1]heptane-1-carboxylate (**8**)

Acetate (*R*)-**1** (50 mg, 0.26 mmol) was treated with a 0.5 M solution of KOH in methanol (5 mL) and stirred for 45 min at room temperature. After this time, water (10 mL) was added, and the solution was extracted with CH_2_Cl_2_ (2 × 10 mL). The combined organic layers were washed with brine (10 mL), dried over MgSO_4,_ and concentrated under reduced pressure. The crude residue was dissolved in CH_2_Cl_2_ (5 mL), and catalytic amounts of 4-dimethylaminopyridine (DMAP), triethylamine (0.1 mL, 0.65 mmol, 2.5 equiv), and (1*S*)-(−)-camphanic chloride (0.4 mmol, 87 mg) were added at room temperature. After 5 h of stirring, a saturated aqueous solution of NH_4_Cl (5 mL) was slowly added, and the mixture was diluted with more CH_2_Cl_2_ (10 mL). The organic layer was successively washed with aqueous 1 M HCl (20 mL), saturated aqueous NaHCO_3_ (20 mL), and brine (20 mL), and dried over MgSO_4_. The crude residue obtained after evaporation of the solvent under reduced pressure was purified by flash column chromatography on silica gel, using a 90:10 mixture of hexane and Et_2_O as eluent, to give 74 mg (85% yield) of camphanate ester **8** as a white solid. This compound was crystallized from cold hexane and was characterized by X-ray crystallography. Spectroscopical data are shown below, for clarity, signals corresponding to the camphanic ring are identified by the abbreviation “camph”: ^1^H NMR (300 MHz, CDCl_3_) (Figure S14) δ 6.08 (1H, s, H-2), 4.99–4.76 (3H, m, CH_2_O and CH’H-3), 4.73–4.66 (1H, m, CHH’-3), 2.52–2.36 (2H, m, H-3 and H_exo_-6 Camph), 2.13–2.00 (1H, m, H_endo_-6 Camph), 1.93 (1H, m, H_exo_-5 Camph), 1.75–1.61 (1H, m, H_endo_-5 Camph), 1.12 (3H, s, Me-4 Camph), 1.10 (3H, s, Me-5α), 1.07 (3H, s, Me-7 Camph), 1.04 (3H, d, *J* = 7.1 Hz, Me-4), 0.98 (3H, s, Me’-7 Camph), 0.90 (3H, s, Me-5β). ^13^C NMR (75 MHz, CDCl_3_) (Figure S15) δ 178.2 (CO_2_ Camph), 167.5 (CO_2_), 156.2 (C-3), 151.8 (C-1), 130.0 (C-2), 103.6 (=CH_2_), 91.2 (C-1 Camph), 62.1 (CH_2_O), 54.9 and 54.3 (C-4 Camph and C-7 Camph), 49.1 (C-4), 47.6 (C-5), 30.9 (C-6 Camph), 29.1 (C-5 Camph), 26.2 (Me-5α), 22.3 (Me-5β), 17.0 (2xMe-7 Camph), 12.4 (Me-4), 9.9 (Me-4 Camph). HRMS (TOF MS ESI^+^) calcd for C_20_H_29_O_4_ [M + H]^•+^ 333.2060, found 333.2069; [α]_D_ −15.5 (c 0.69, CHCl_3_); mp 70.4–71.2 °C.

### Field Activity Bioassay

The response of *D. aberiae* males to the different synthetic enantiomers and their mixture was evaluated under field conditions. Five blocks of four traps were installed in a citrus orchard (*Citrus reticulata* cv. Clemenules), located in the municipality of Vila-real (Castellón, Spain) to test the attractant activity of (−)-(*R*)-**1**, (+)-(*S*)-**1**, and 1:1 mixture of (−)-(*R*)-**1** and (+)-(*S*)-**1**. A fourth trap without any attractant was also included in the test (blank). White sticky cardboard traps (95 mm × 150 mm; Ecología y Protección Agrícola SL, Carlet, Spain) were baited with rubber septa (Ecología y Protección Agrícola SL, Carlet, Spain) dispensers, loaded by immersion with the corresponding hexane solutions of the test substances (100 μg of (−)-(*R*)-**1**, 100 μg of (+)-(*S*)-**1** or 200 μg of racemic mixture (1:1, (−)-(*R*)-**1**: (+)-(*S*)-**1**)). The purity of the individual enantiomers was (>97% ee, see Results and Discussion section). The sticky cardboards were replaced weekly and taken to the laboratory to count the number of males captured per trap and week (MTW) under a stereomicroscope (Stemi 508; Zeiss, Oberkochen, Germany). Traps were rotated weekly within each block for 3 weeks and the dispensers were not replaced throughout the experiment. Traps were hung on tree branches at a height of 1.5 m and were spaced 20 m apart, with each block at least 50 m apart.

Generalized linear mixed models (GLMM) were constructed to assess the significance of the differences observed among the different substances (treatments) tested, using R version 4.0.3 (The R Foundation for Statistical Computing 2020). For this purpose, the *glmer* function from the *lme4* package was employed by assuming the poisson error distribution. Models were constructed with the number of males captured per trap and week (MTW) as the dependent variable, treatment, time (week of the study period) and their interaction (treatment × time) as fixed factors, and block (experimental replicate) as random factor. The significance of the different effects was assessed by removing the corresponding factor from the model and comparing models with likelihood ratio tests. The *glht* function in the multcomp package was then used to perform Tukey HSD tests for post hoc pairwise comparisons.

## Results and Discussion

The synthetic strategy followed for the preparation of both enantiomers of **1**, (*R*)-**1** and (*S*)-**1**, is depicted in the retrosynthetic scheme shown in Figure 2. The key intermediate of these syntheses is the alcohol **ii**, a mixture of diastereomers, epimers at C–4. Each of the enantiomers of **1** could be prepared from the corresponding diastereomer of **ii**, after their chromatographic separation, via oxidation of the allylic hydroxyl group, methylenation of the resulting carbonyl group, deprotection of the hydroxyl methyl group, and acetylation. Intermediate **ii**, with the necessary configuration at the secondary carbinolic center, could be obtained from cyclopentenone **i** through diastereoselective reduction of the carbonyl group using Corey-Bakshi-Shibata (CBS) reduction conditions.^15^ A cyclopentenone such as **i** can be readily synthesized in the racemic form from isobutyl (*E*)-but-2-enoate (**2**) following the synthetic strategy previously described for the preparation of this cyclopentenone framework.^7^

Based on the above retrosynthetic analysis, the synthesis of enantiomers (*R*)-**1** and (*S*)-**1** began with the cyclization of isobutyl (*E*)-but-2-enoate (**2**) promoted by polyphosphoric acid, under the conditions previously reported by Conia and Leriverend,^14^ to obtain cyclopentenone **3** (Figure 3). Enone **3** was photochemically brominated at the allylic position using *N*-bromosuccinimide in CH_2_Cl_2_, yielding the brominated derivative **4** with a 60% yield.^16,17^ Bromo ketone **4** was then transformed into pivaloyl ester **5** in 85% yield via nucleophilic substitution reaction of the bromine atom with potassium pivalate in *tert*-butyl methyl ether. Then, **5** was regioselectively methylated at C–4, using lithium bis(trimethylsilyl)amide as a base at −30 °C and methyl iodide as methylating reagent, to give racemic methyl ketone **6** in a moderate 62% yield (Figure 3), which was significantly higher than that previously obtained in the methylation reaction of the analogous acetylated derivative.^7^

Diastereoselective reduction of cyclopentenone **6** to an equimolecular mixture of diastereomeric cyclopentenols **7** was accomplished using borane-dimethylsulfide complex (BH_3_·SMe_2_) in the presence of (*S*)-(−)-2-methyl-CBS-oxazaborolidine at −40 °C (Figure 4).^18^ Both diastereomers could be easily separated by flash column chromatography, providing (3*R*,4*S*)-**7**, the less polar diastereomer, and (3*R*,4*R*)-**7**, the more polar diastereomer, in a combined yield of 86%. The different polarity of both diastereomers could be rationalized based on the lower hydrogen bond formation capacity of the hydroxyl group at C-3 of the less polar diastereomer, due to the greater steric hindrance.

The configuration of the new stereogenic center generated at C–3 of each diastereomer was tentatively assigned as (*R*), based on the chiral oxazaborolidine used as catalyst, which directs the hydride transfer reaction from the reface of the carbonyl group.^19^ On the other hand, the relative stereochemistry of each diastereomer was established based on their spectroscopic data and, particularly, NOE (nuclear Overhauser effect) experiments. Thus, a *cis*-relationship of the methyl and hydroxyl groups at C–3 and C–4, respectively, could be inferred from the ^13^C NMR of the less polar diastereomer [(3*R*,4*S*)-**7**] which showed a significant upfield-shifted chemical shift of the Me group at C–4 (δ_C_ 8.4 ppm compared to δ_C_ 11.3 ppm in the more polar diastereomer), due to the γ-effect exerted by the hydroxyl group. Additionally, the assigned stereochemistry to the more polar diastereomer [(3*R*,4*R*)-**7**] also agreed with the results of NOE experiments in which irradiation of the signal at δ_H_ 4.40–4.25 ppm (the α–oriented H–3) gave enhancement of the signal at δ_H_ 1.06 ppm, which corresponds to the α–oriented methyl group at C–4. (Figures S16–S17).

**Figure 5:**
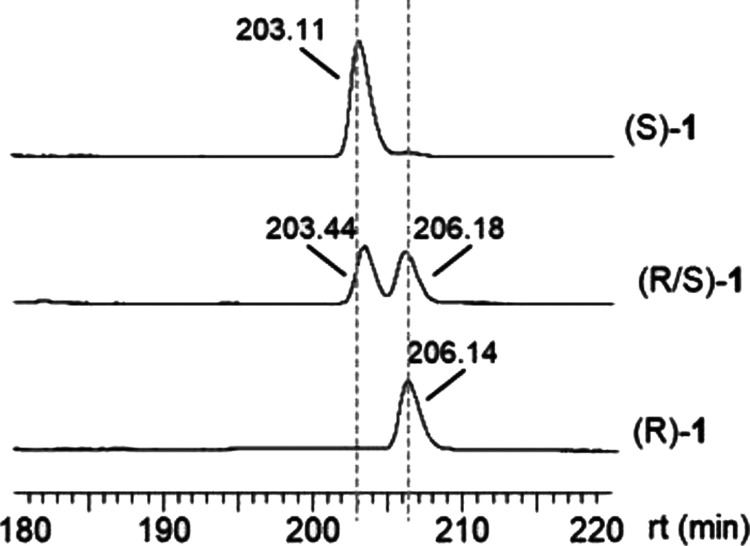
Chiral GC chromatogram of single (*R*)–**1**, (*S*)–**1** and coeluted samples.

Finally, both diastereomeric alcohols, (3*R*,4*S*)-**7** and (3*R*,4*R*)-**7**, were individually transformed to the enantiomers of the sex pheromone, (*R*)**-1** and (*S*)**-1**, respectively, through a series of functional group transformations (Figure 4). First, oxidation of the secondary alcohol with pyridine chlorochromate (PCC) afforded enantiomeric ketones (*R*)**-6** and (*S*)**-6** in 82–88% yield.^20^ Methylenation of the unsaturated carbonyl moiety using Petasis’s reagent,^21^ followed by *in situ* consecutive treatment of the reaction mixture with MeMgCl and EtOAc, promotes the transformation of the pivalate ester moiety into the acetate one, affording enantiomers (*R*)**-1** and (*S*)**-1** with an overall yield of about 58–60% for the three synthetic transformations involved in this *one pot* transformation. An enantiomeric excess higher than 97% was determined for both enantiomers by gas chromatography (GC) with a chiral stationary phase column (Figure 5). The specific rotation of both enantiomers was measured and (*R*)-**1** was identified as levorotatory and (*S*)-**1** as dextrorotatory. Given that Vacas et al.^7^ assigned the levorotatory enantiomer to the natural *D. aberiae* sex pheromone, we established its absolute configuration as that showed in structure (−)-(*R*)**-1**.

**Figure 6:**
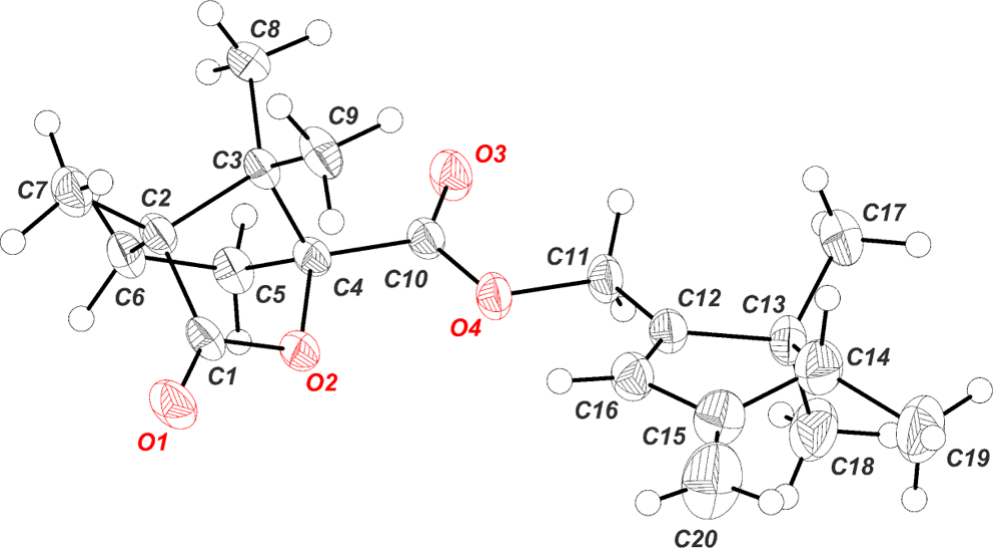
ORTEP diagram for the camphanic acid derivative of (*R*)–**1** (compound **8**) Thermal ellipsoids are shown with 50% of probability. For details, see CCDC 2327985 (Cambridge Crystallographic Data Centre).

Definitive confirmatory evidence of the absolute configuration of the stereocenter present in the natural sex pheromone emitted by *D. aberiae* virgin females was obtained after an X-ray diffraction analysis was performed on a crystalline derivative of enantiomer (−)-(*R*)-**1**. The crystalline sample was prepared by hydrolysis of the acetate moiety of this enantiomer with KOH in methanol, followed by direct esterification of the crude material with (1*S*)-(−)-camphanic chloride and Et_3_N in CH_2_Cl_2_. Purification of the sample by flash column chromatography afforded the camphanate ester **8** as a solid with an overall yield of 85%. An appropriate sample for single crystal X-ray analysis was obtained by crystallization in cold hexane, whose ORTEP diagram is shown in Figure 6, confirming the absolute configuration initially proposed for the stereogenic center at C–4.

**Figure 7:**
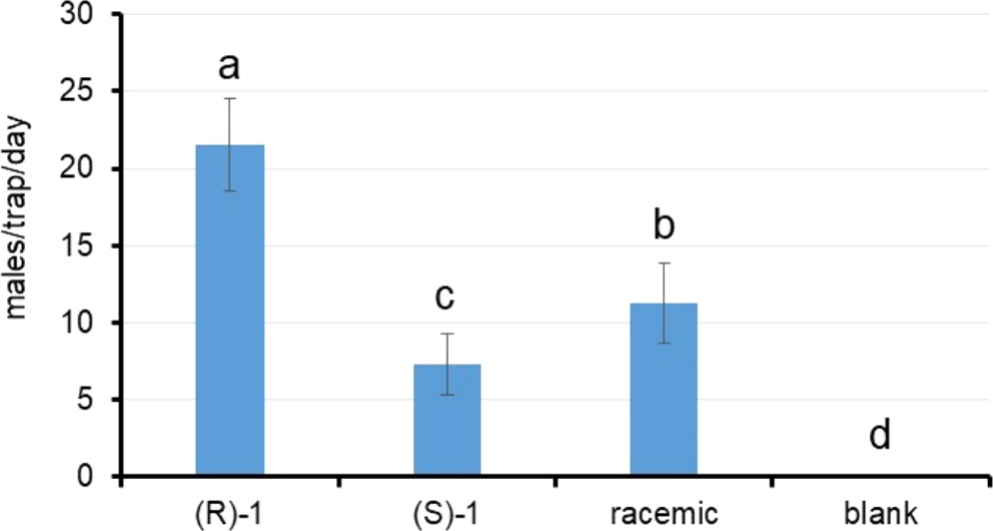
Mean (±SE) number of males per trap and week captured with the different enantiomers, the racemic mixture and the blank. Bars labeled with different letters were significantly different (Tukey HSD tests, at *P* < 0.05).

Once obtained, the attractant activity of both synthetic enantiomers was tested under field conditions. Traps baited with the natural (−)-(*R*)-**1** and the non-natural (+)-(*S*)-**1** enantiomers captured a total of 2831 and 914 males, respectively, whereas 1481 males were recorded with the racemate and only 18 in the blank traps (Figure 7). The statistical analysis revealed that treatment had a significant effect on male catches (χ^2^ = 4041.3; *P* < 0.0001), with (−)-(*R*)-**1** achieving significantly higher trapping efficacy than both (+)-(*S*)-**1** and the racemic mixture. The factor time also had significant effects on captures (χ^2^ = 1189.7; *P* < 0.0001), due to the fluctuating population levels of the pest during the weeks of study. The effect of the interaction treatment × time also resulted significant (χ^2^ = 187.3; *P* < 0.0001), probably because weekly fluctuations in population levels are not detected in the same way for the different treatments.

Interestingly, the racemic mixture displayed significantly lower efficacy than pure (−)-(*R*)-**1** but significantly higher than (+)-(*S*)-**1** (Figure 7). Given that mean captures in traps without attractant (blank) were 0.1 males/trap/day, our data shows that (+)-(*S*)-**1** enantiomer obtained not negligible trap catches despite being the opposite enantiomer to the natural. These captures could be partially explained by the residual presence of the natural enantiomer (*R*)-**1** in the synthesized sample. However, at the enantiomeric ratio tested (50:50) in our trial, capture data suggest that the presence of (*S*)-**1** had a detrimental effect on the attraction of the mixture, as seen in the significance of the differences (Figure 7). This adds a new example confirming that bioactivity depends on the chirality of the pheromones, extensively reported in the literature reviewed by Mori.^13−22^ This kind of detrimental effect has been reported for the sex pheromones of the gypsy moth (*Lymantria dispar* L.), proving that the unnatural (−)-enantiomer drastically reduced the response of the moths to the naturally occurring (+)-enantiomer; and similar for the Japanese beetle (*Popillia japonica* Newman), with the (*S*,*Z*)-isomer causing a strong inhibition over the activity of the natural (*R*,*Z*).^22^ However, this detrimental relationship has never been described for mealybugs, for which only a single enantiomer is usually bioactive, and its opposite enantiomer does not inhibit the response to the active stereoisomer, as reported for the vine mealybug (*Planococcus ficus* Signoret), citrus mealybug (*Pseudococcus cryptus* Hempel) and pink hibiscus mealybug (*Maconellicoccus hirsutus* (Green)).^13^

In the present research, a synthesis of both enantiomers of (4,5,5-trimethyl-3-methylenecyclopent-1-en-1-yl)methyl acetate has been achieved via diastereomeric resolution of secondary alcohols obtained from the chiral reduction of ketone **6** (Figure 4) under Corey-Bakshi-Shibata conditions. This has allowed to unequivocally determined the absolute configuration of the sex pheromone in the species *D. aberiae* as (−)-*R*-(4,5,5-trimethyl-3-methylenecyclopent-1-en-1-yl)methyl acetate. Interestingly, field trials have revealed a significantly lower activity of the non-natural (*S*)-**1** enantiomer and a detrimental effect caused by its presence when using a racemate. This finding may have practical consequences showing that a deeper understanding of the species’ behavior is needed for future research aimed at maximizing the efficacy of the monitoring and control strategies for this invasive mealybug.

## Supporting Information Available

^1^H NMR and ^13^C NMR spectra of the synthetic compounds (Figures S1–S15); NOE NMR spectrum of (3*R*,4*S*)-**7** and (3*R*,4*R*)-**7** (Figures S16 and S17); chromatography (GC) with a chiral stationary phase column of (3*R*,4*S*)-**7** and (3*R*,4*R*)-**7** (Figure S18), and crystal data and structure refinement for compound **8** (PDF)
