# Supplementary material for: Absolute Configuration of the Invasive Mealybug Delottococcus aberiae (De Lotto) Sex Pheromone: Synthesis and Bioassay of Both Enantiomers
Source: J Agric Food Chem. 2024 Sep 19;72(39):21488–94. doi: 10.1021/acs.jafc.4c05469 (PMC11450821; doi:10.1021/acs.jafc.4c05469)
Supplement: Supplementary file 1 — jf4c05469_si_001.pdf [file jf4c05469_si_001.pdf]

Supporting Information prepared for Journal of Agricultural and Food Chemistry

Absolute configuration of the invasive mealybug *Delottococcus aberiae* (De Lotto) sex pheromone: synthesis and bioassay of both enantiomers

Javier Marzo Bargues,[1,2] Sandra Vacas,[3] Ismael Navarro Fuertes,[2] Jaime Primo,[3] Antonio Abad-Somovilla,[2] Vicente Navarro-Llopis,[3]

[1] Ecología y Protección Agrícola SL, Pol. Ind. Ciutat de Carlet, 46240 Carlet (Valencia), Spain. [2] Universitat de València, Departamento de Química Orgánica, Dr Moliner 50, 46100-Burjassot (Valencia), Spain. [3] CEQA-Instituto Agroforestal del Mediterráneo, Universitat Politècnica de València, Camino de Vera s/n, edificio 6C-5ª planta, 46022 Valencia (Valencia), Spain.

Supporting Information

Figures S1-S15: ^{1}H NMR and ^{13}C NMR spectra of the synthetic compounds.

Figures S16-S17: NOE NMR spectrum of (3R,4S)-**7** and (3R,4R)-**7**.

Figure S18: Chromatography (GC) with a chiral stationary phase column of (3R,4S)-**7** and (3R,4R)-**7**.

Crystal data and structure refinement for compound 8.

Figure S1. ^{1}H NMR spectrum of **5**.

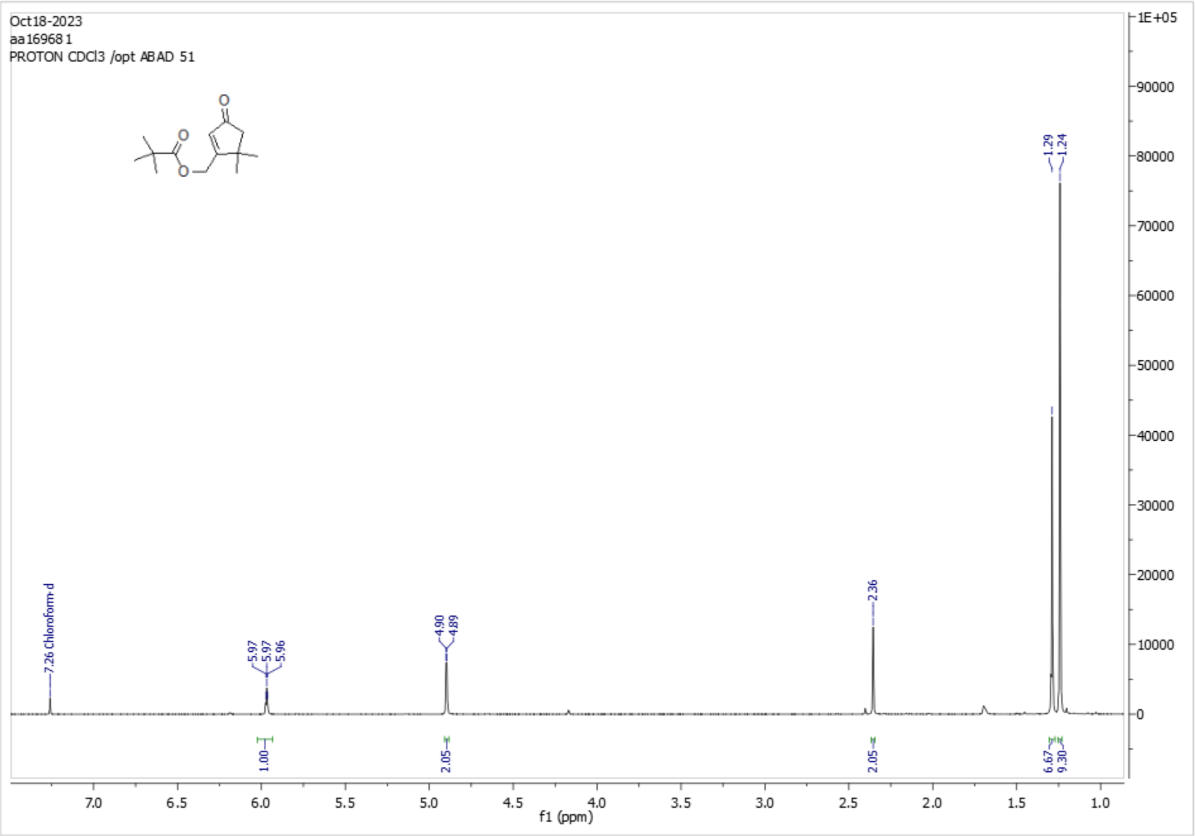

Figure S2. ^{13}C NMR spectrum of **5**.

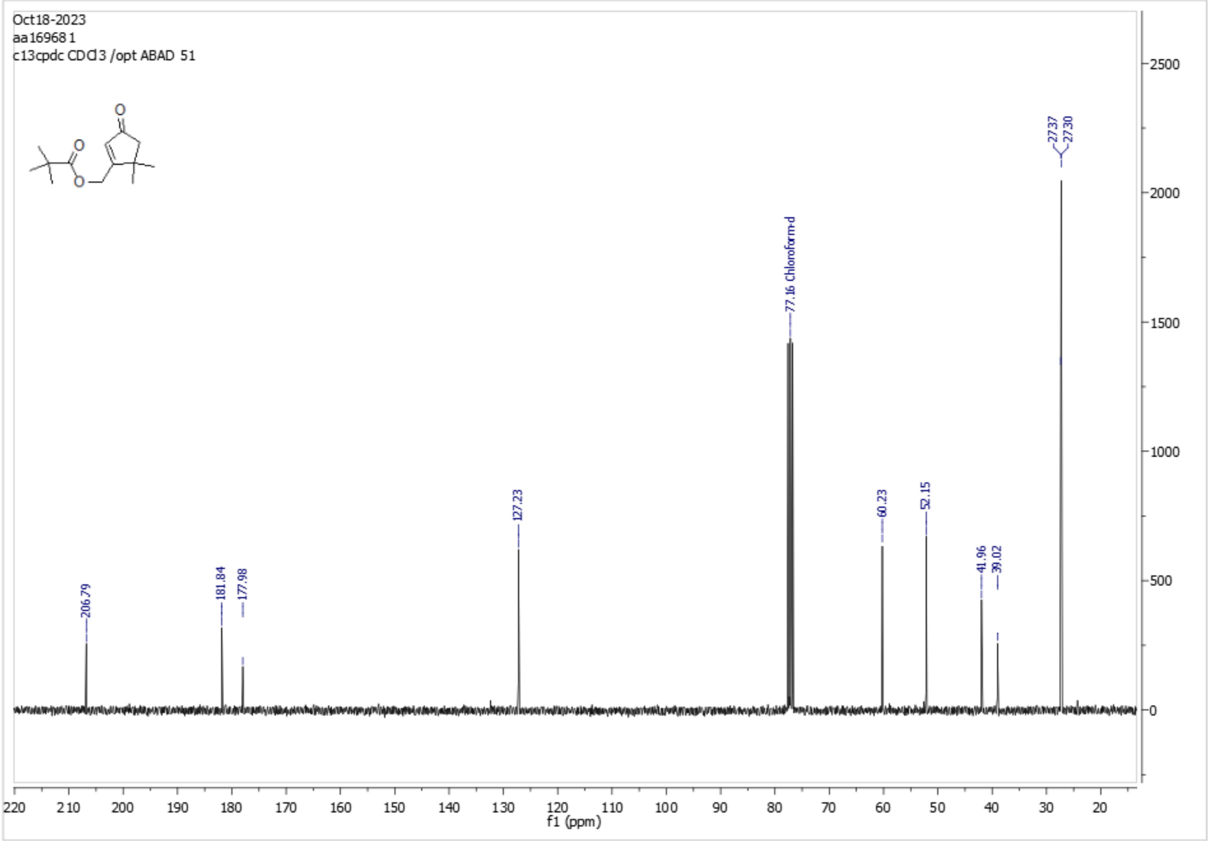

Figure S3. ^{1}H NMR spectrum of **6**.

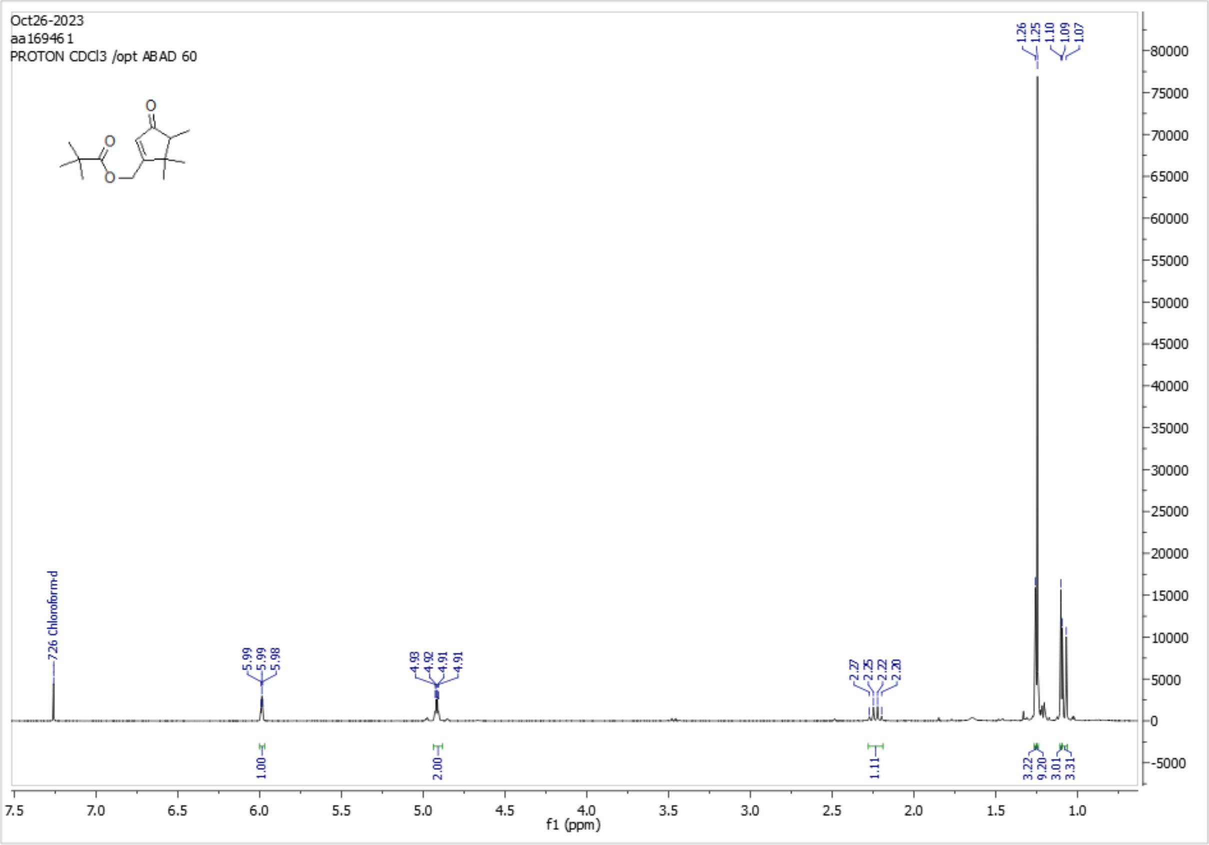

Figure S4. ^{13}C NMR spectrum of **6**.

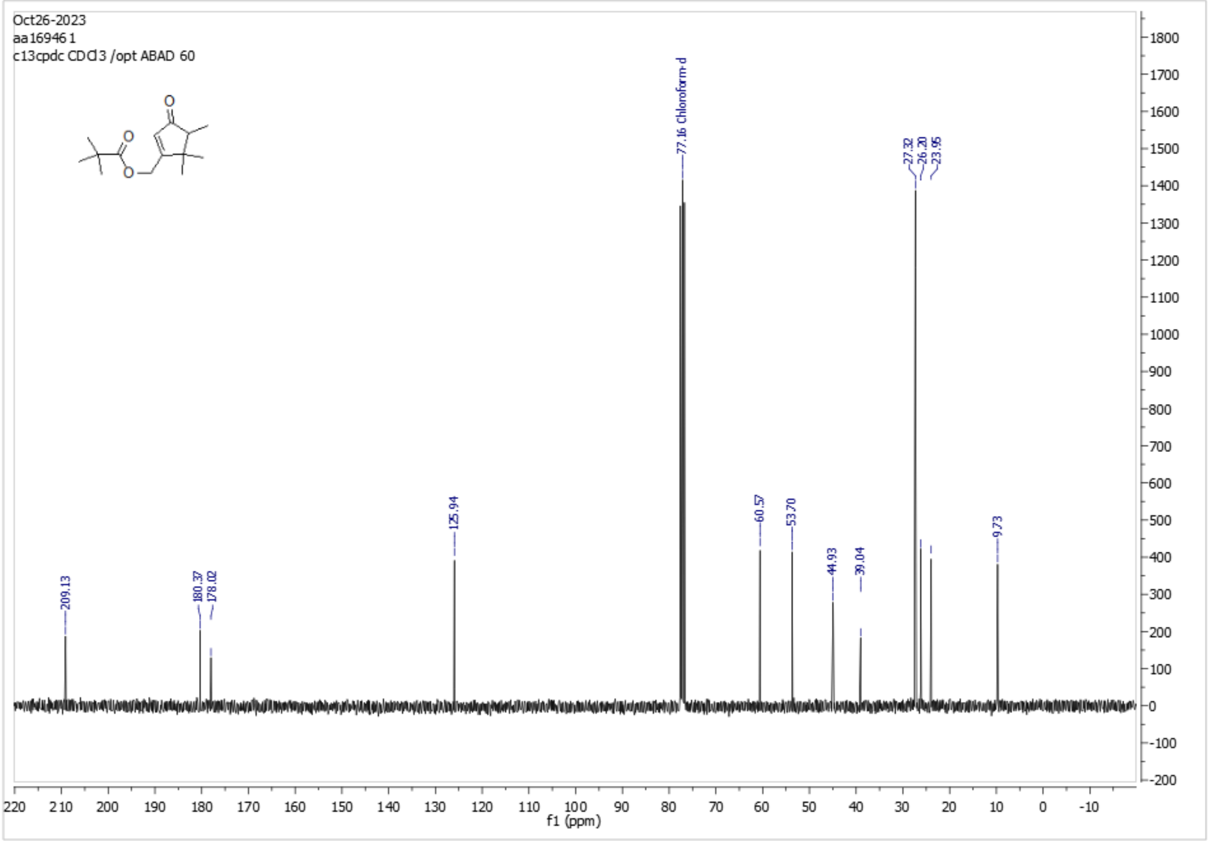

Figure S5. ^{1}H NMR spectrum of **(3R,4S)-7**.

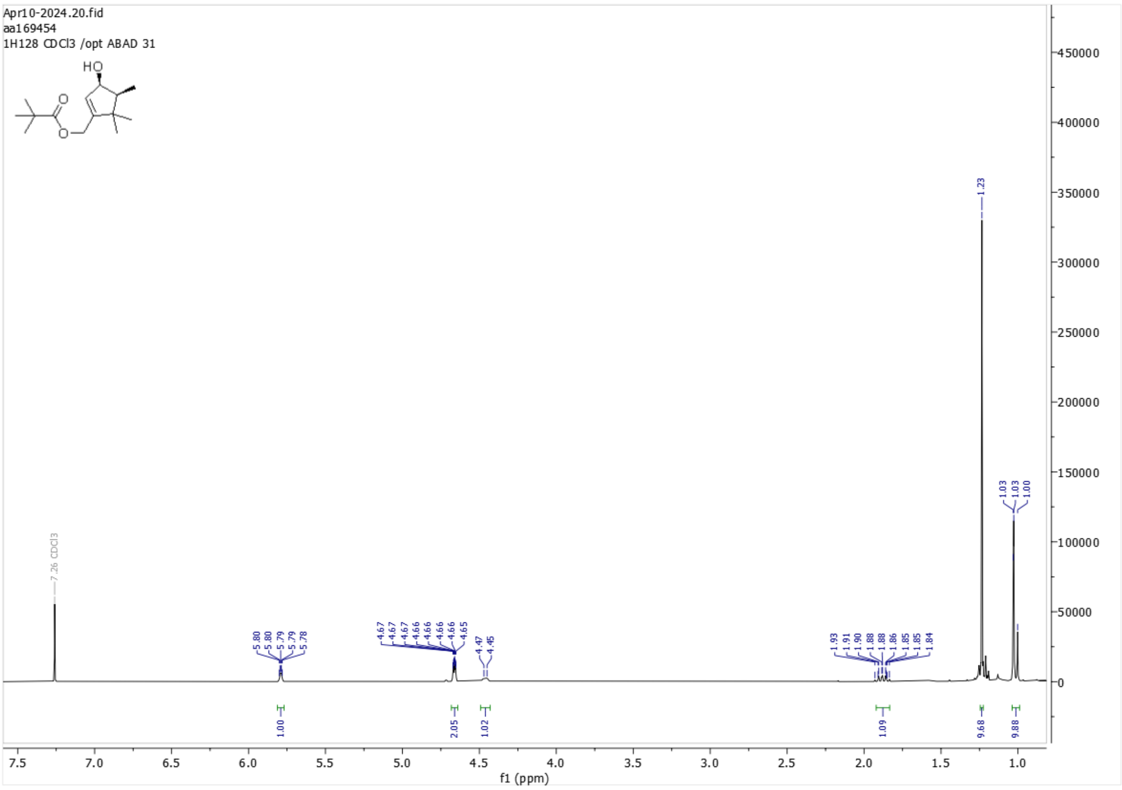

Figure S6. ^{13}C NMR spectrum of **(3R,4S)-7**.

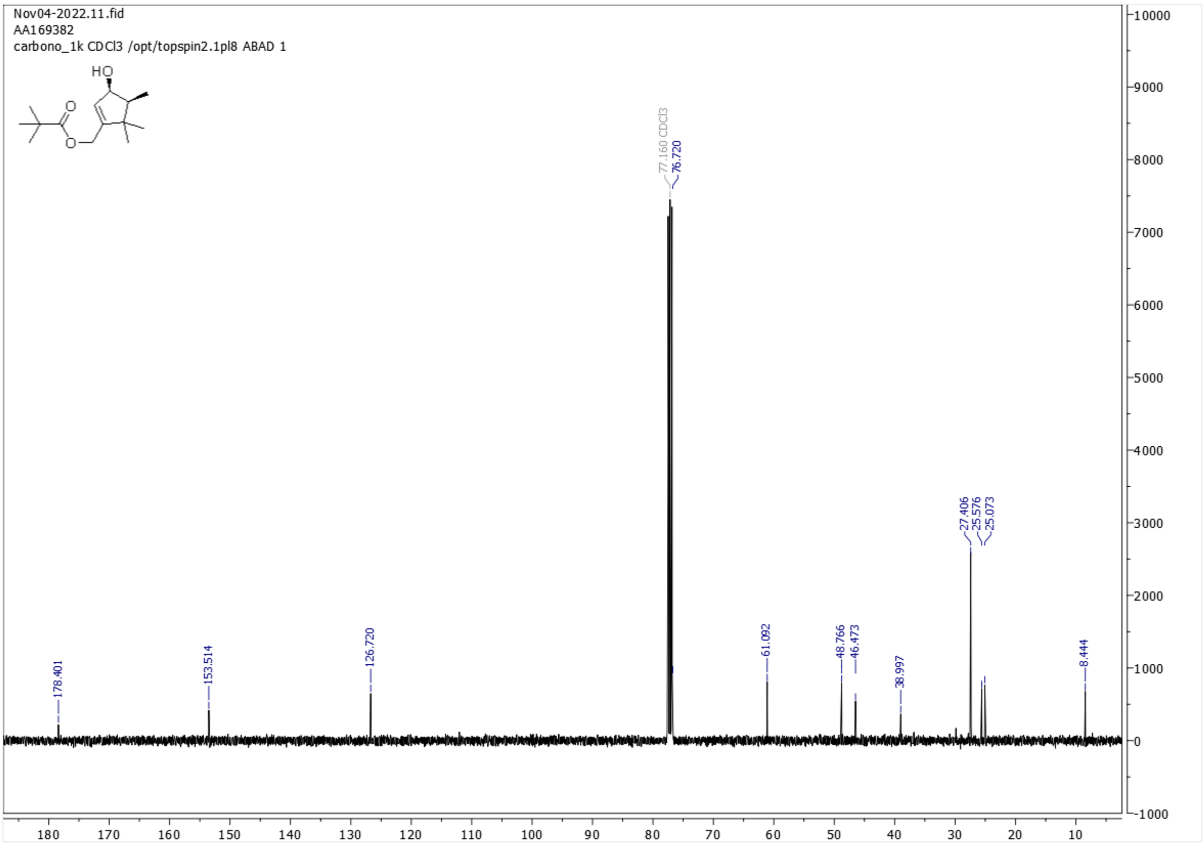

Figure S7. ^{1}H NMR spectrum of **(3R,4R)-7**.

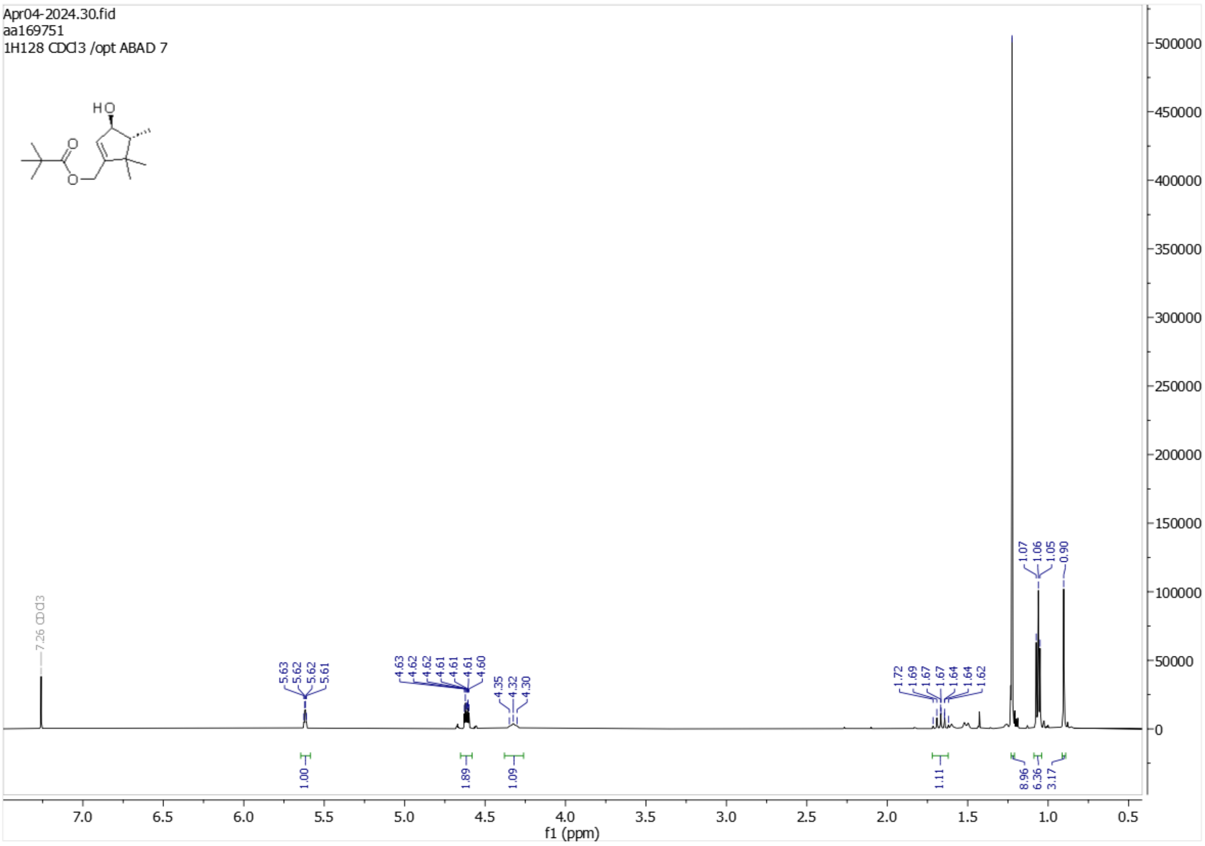

Figure S8. ^{13}C NMR spectrum of **(3R,4R)-7**.

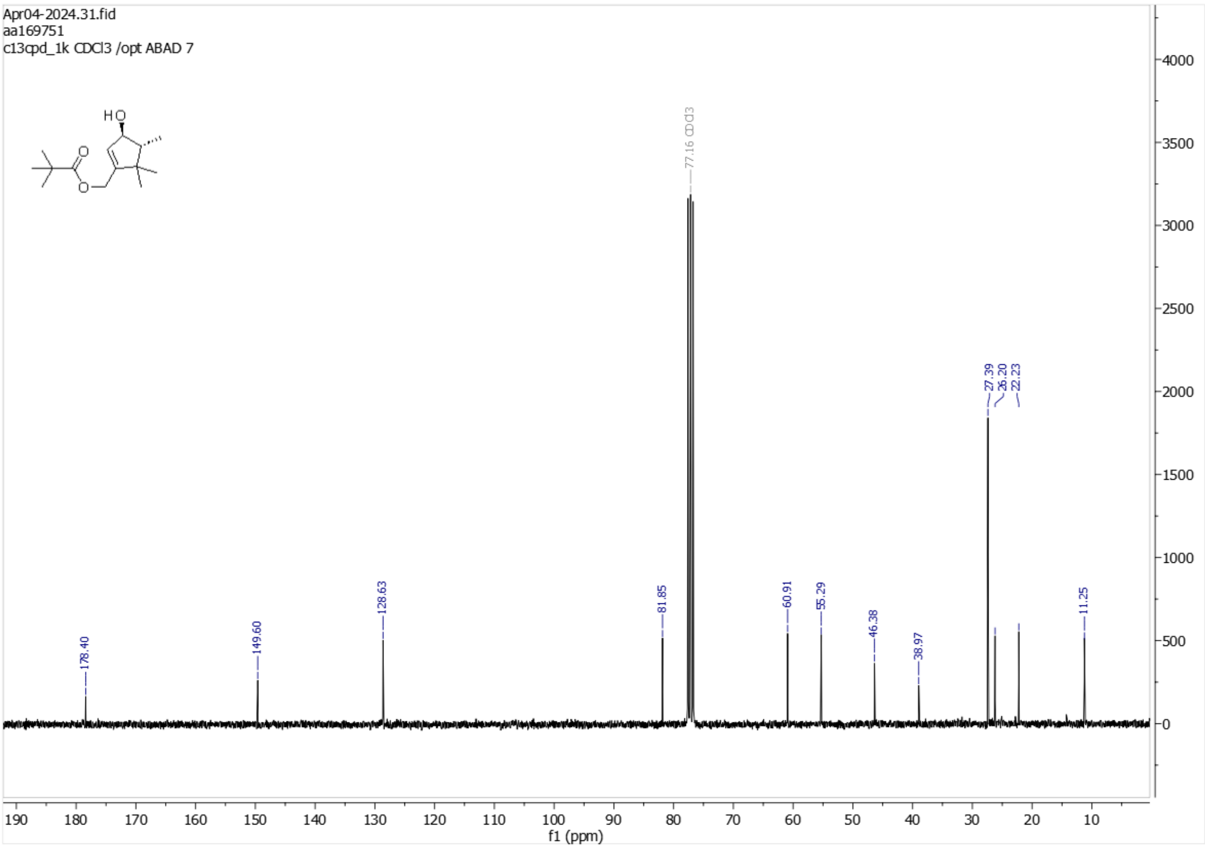

Figure S9. ^{1}H NMR spectrum of **(S)-6**.

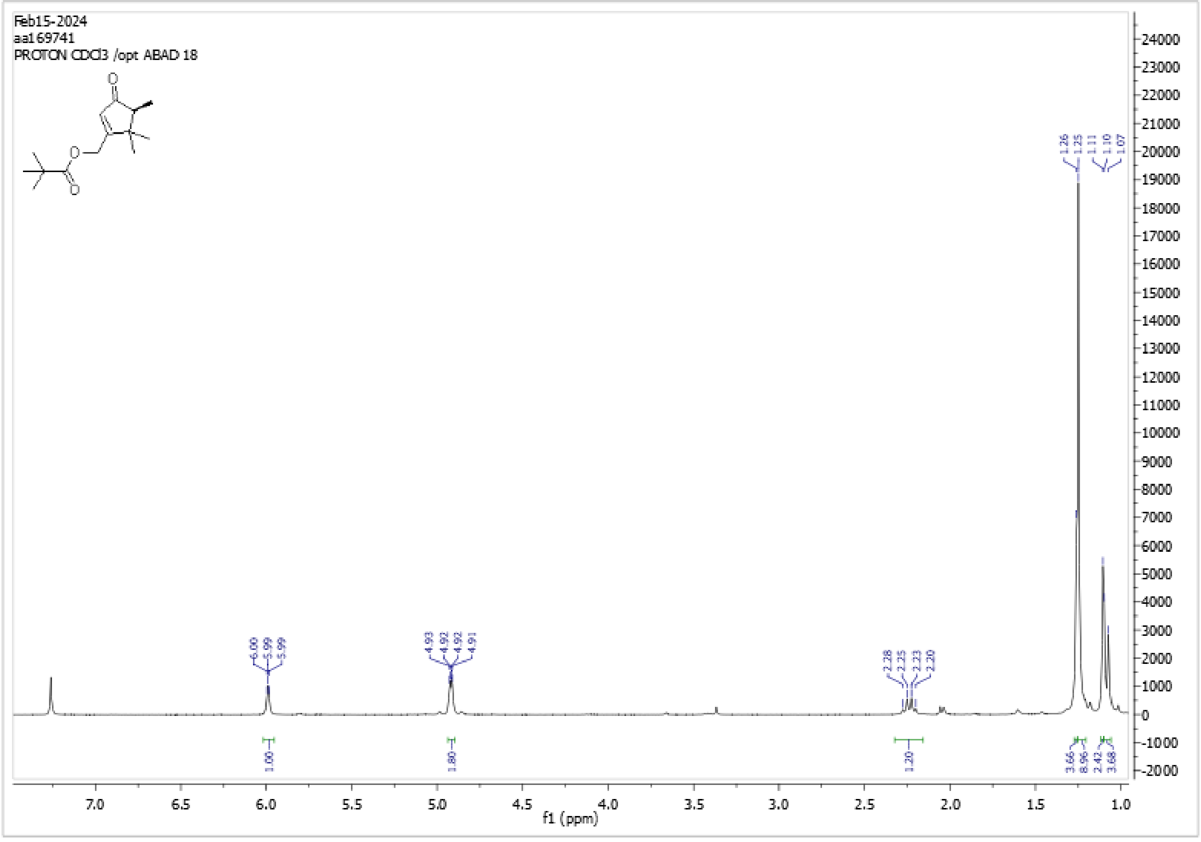

Figure S10. ^{1}H NMR spectrum of **(R)-6**.

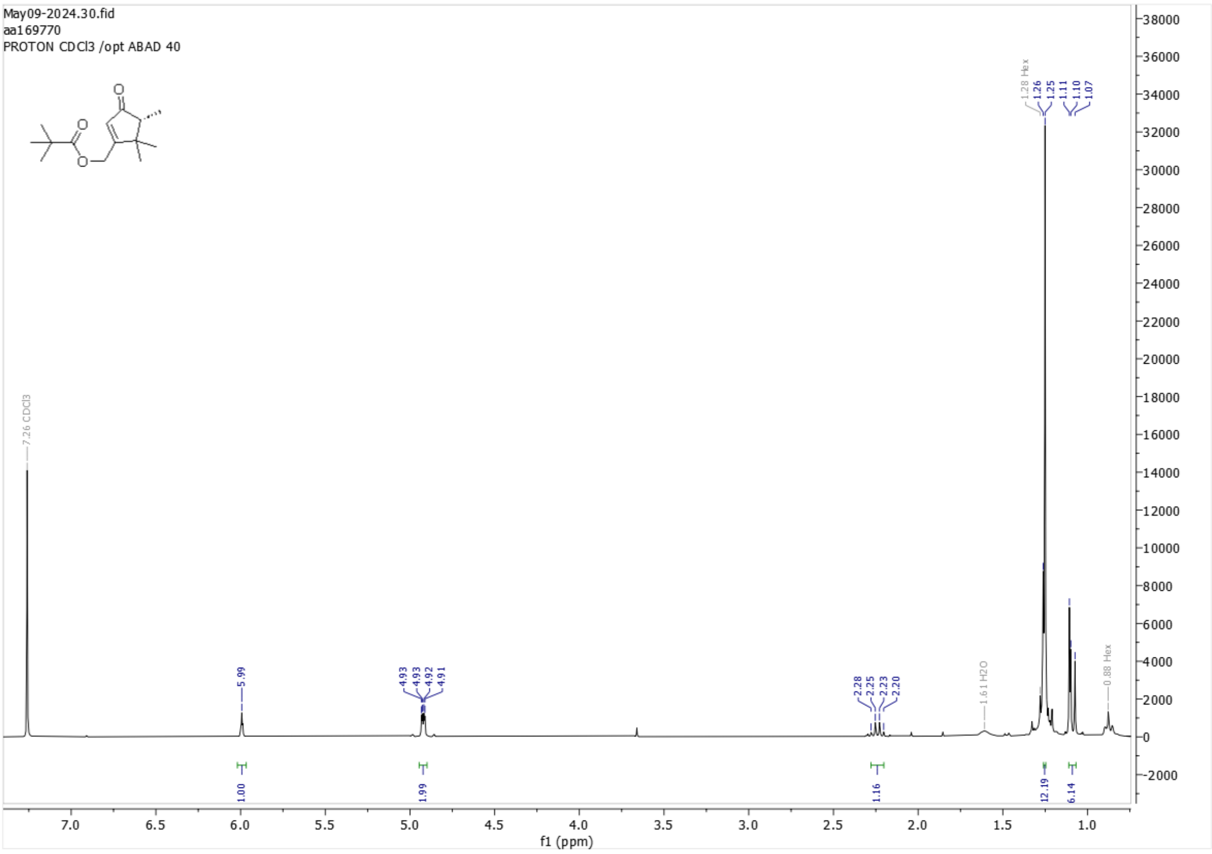

Figure S11. ^{1}H NMR spectrum of **(R)-1**.

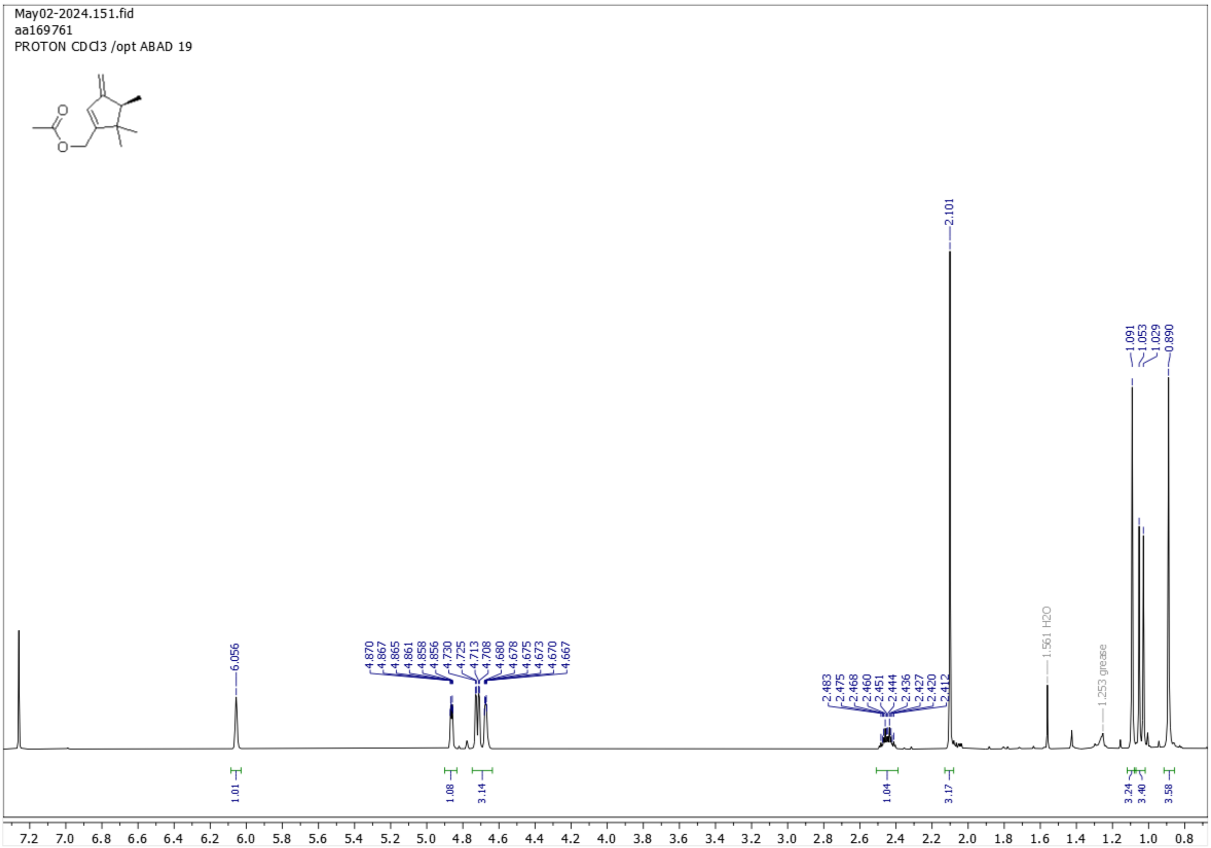

Figure S12. ^{13}C NMR spectrum of **(R)-1**.

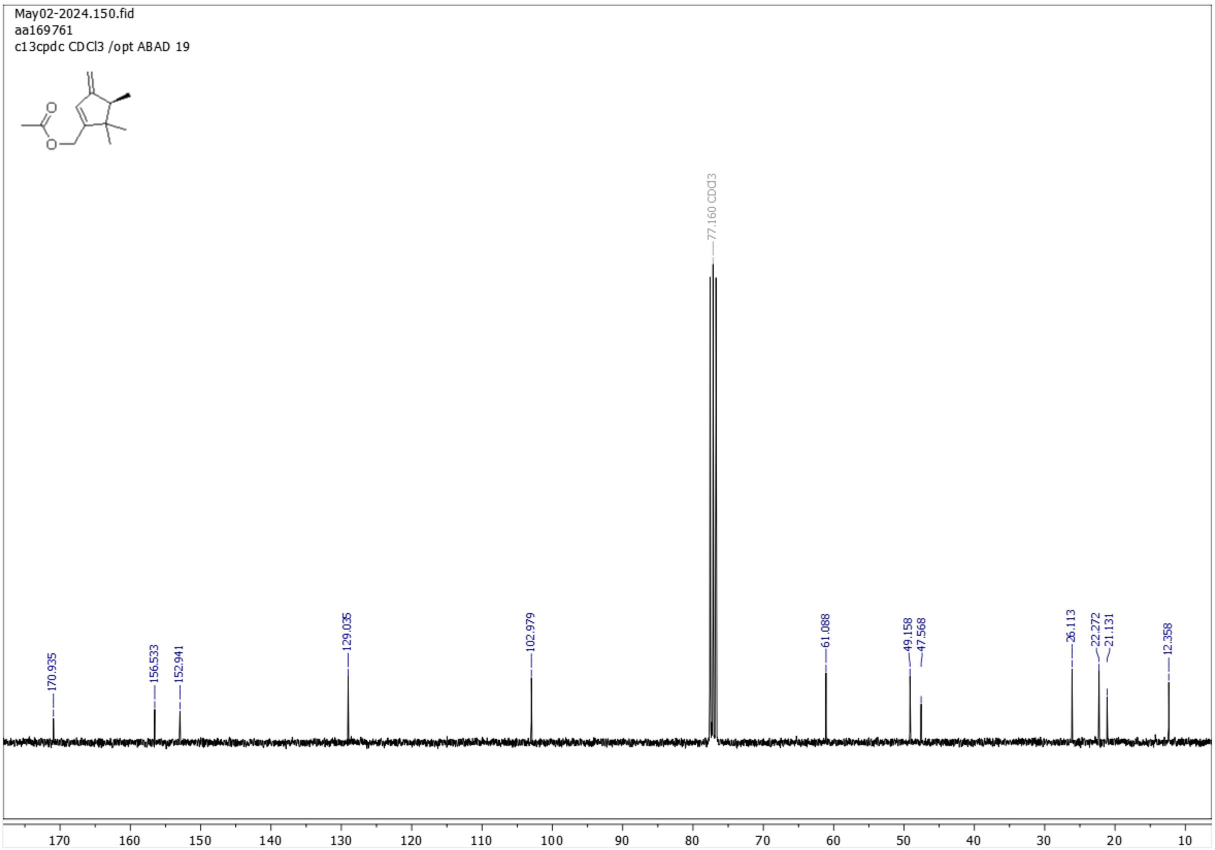

Figure S13. ^{1}H NMR spectrum of **(S)-1**.

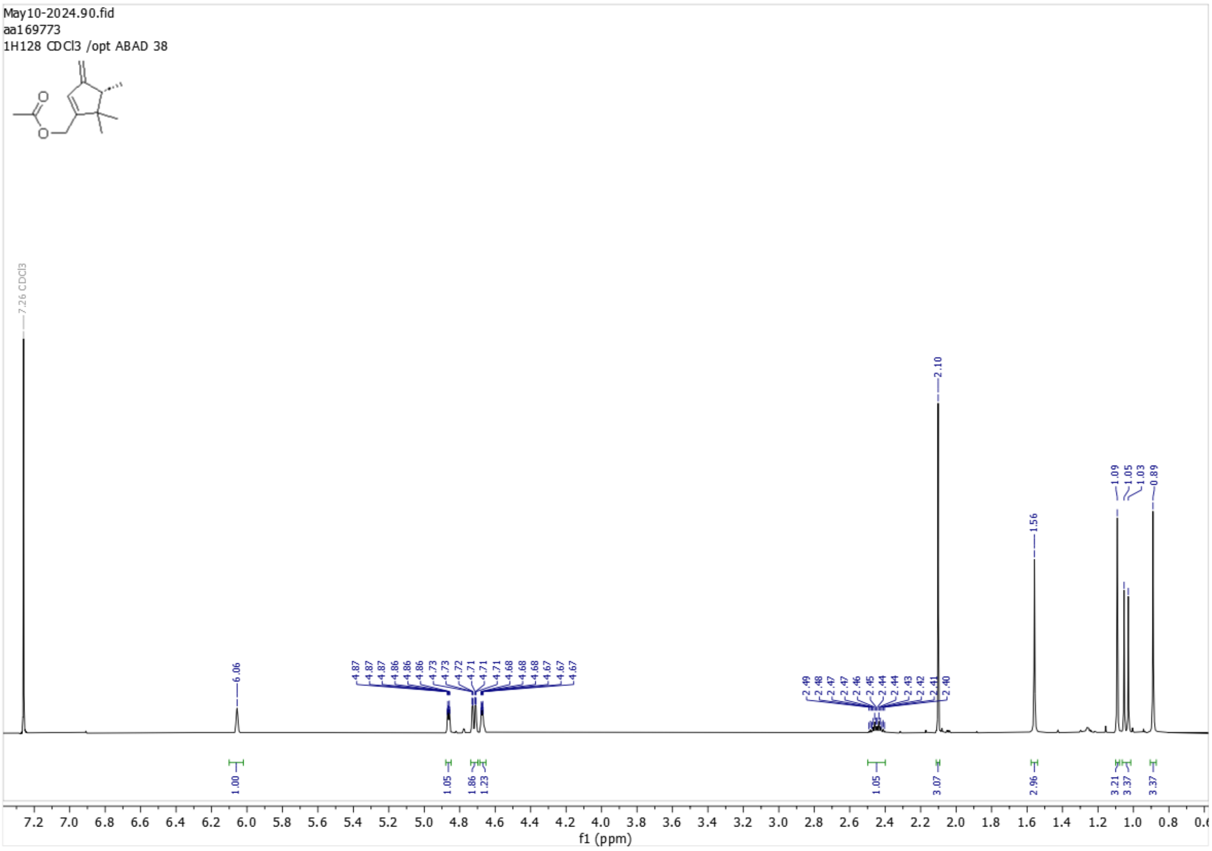

Figure S14. ^{1}H NMR spectrum of **8**.

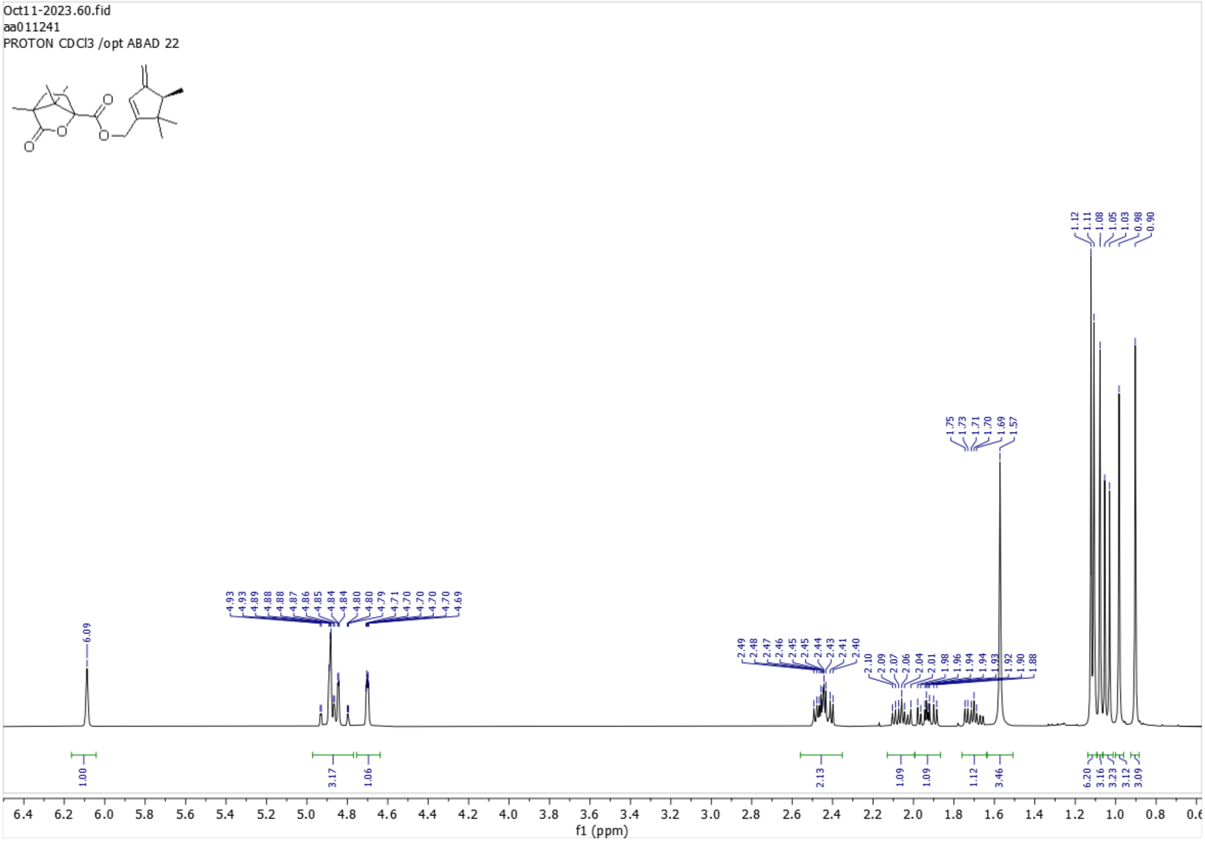

Figure S15. ^{13}C NMR spectrum of **8**.

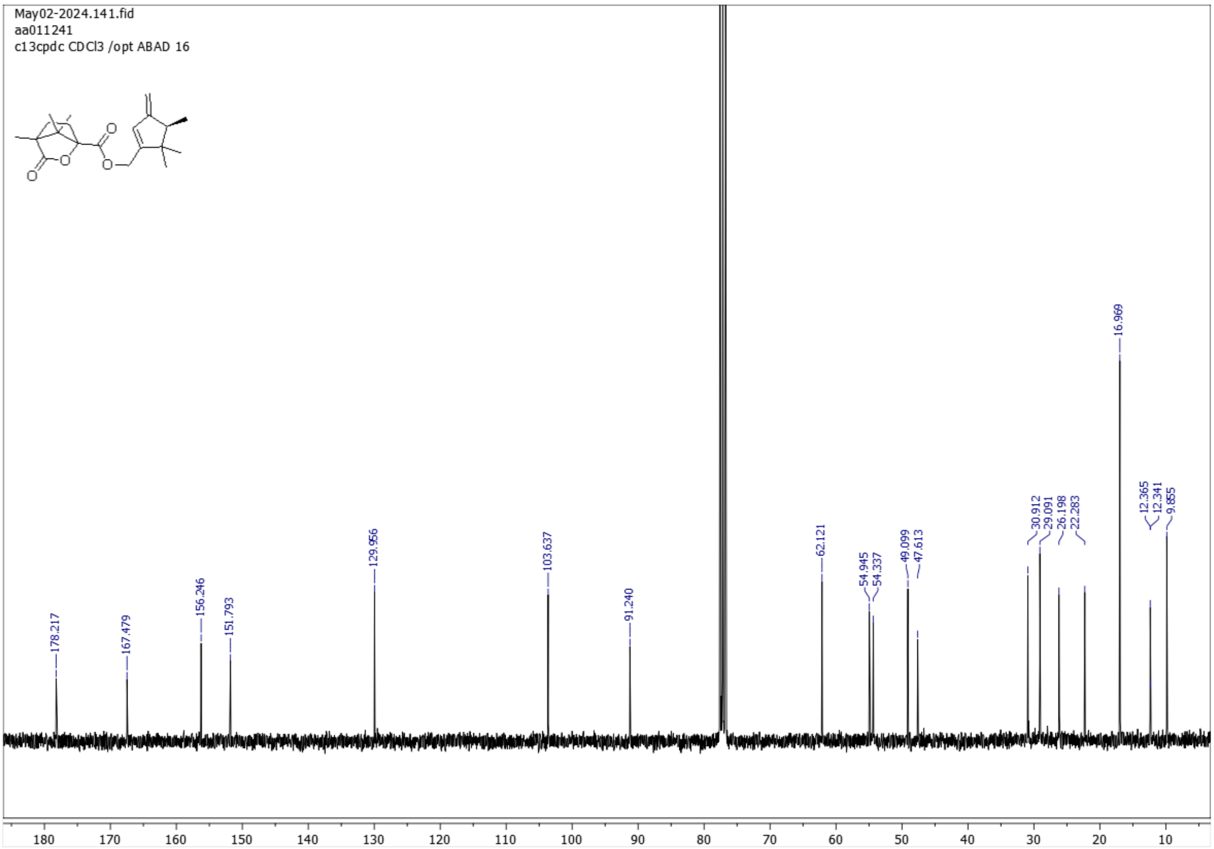

Figure S16. NOE NMR spectrum of **(3R,4R)-7**.

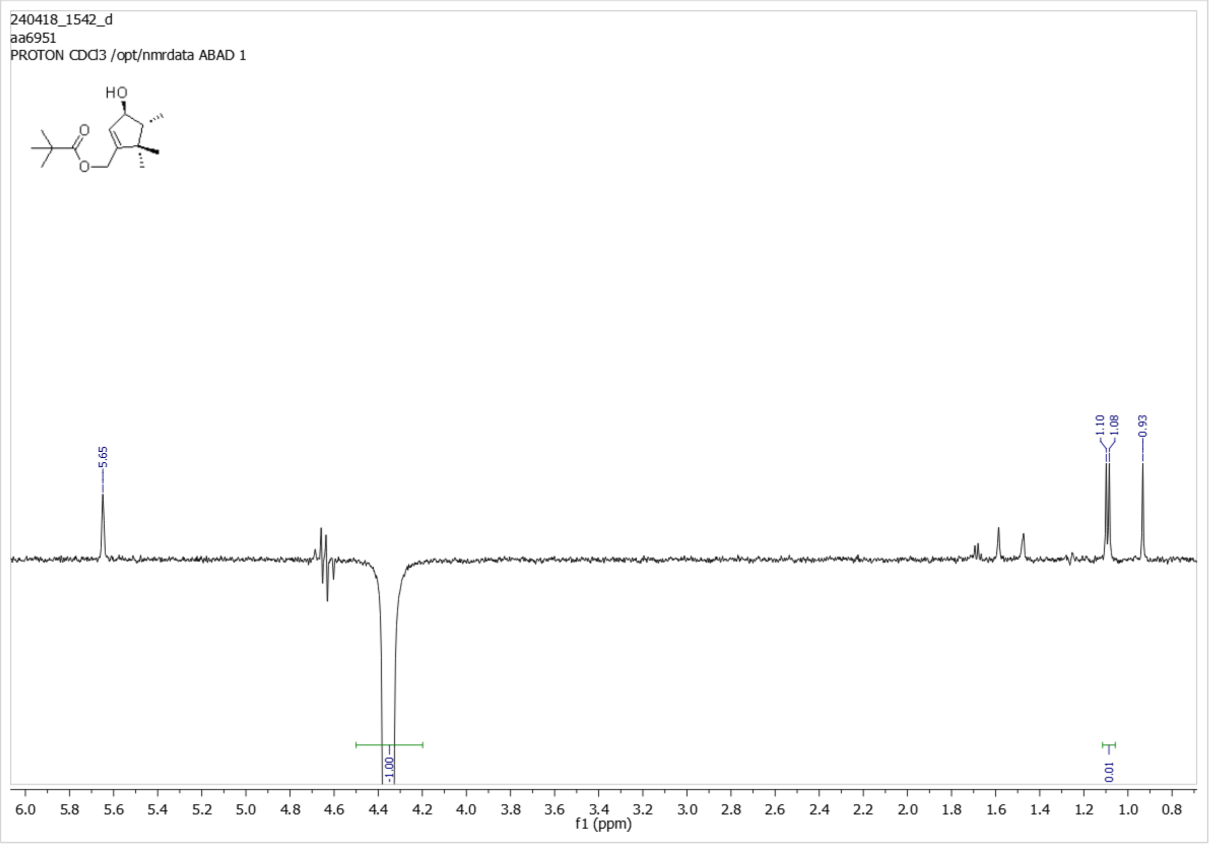

Figure S17. NOE NMR spectrum of **(3R,4S)-7**.

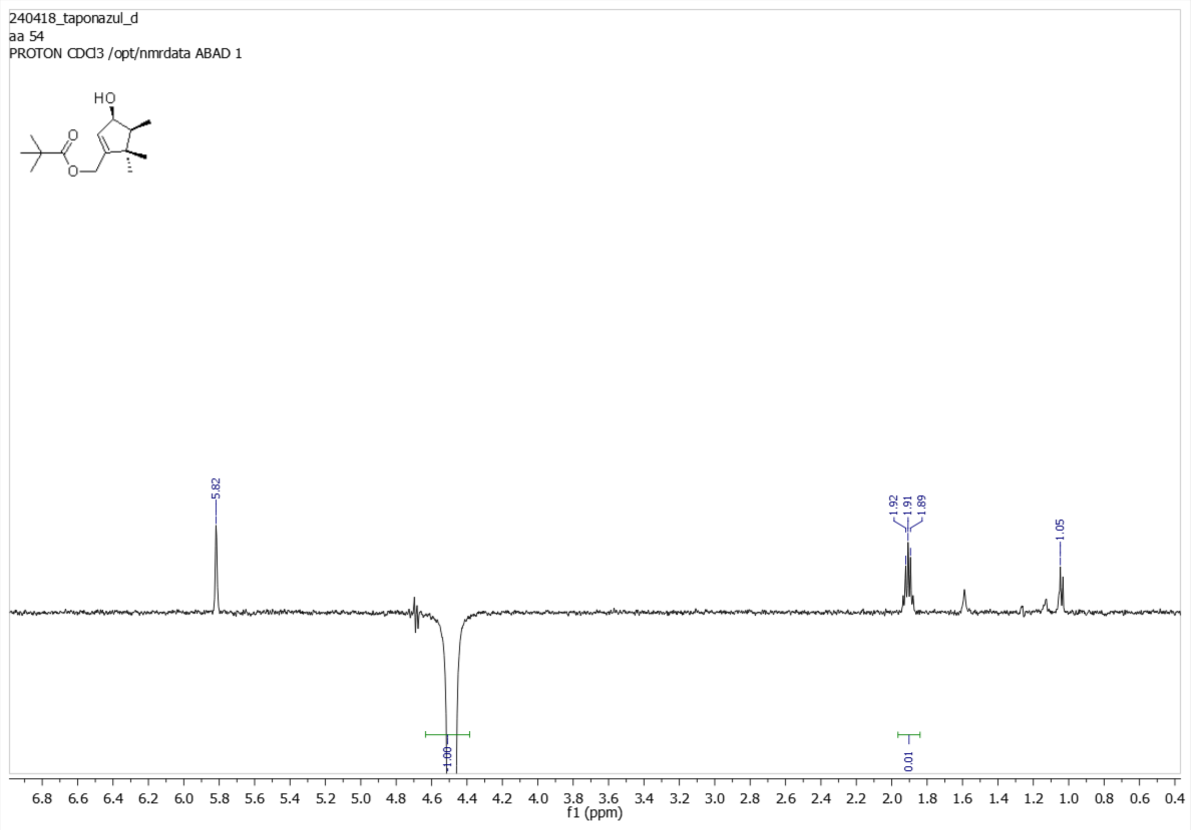

Figure S18: Chromatography (GC) with a chiral stationary phase column of **(3R,4S)-7** and **(3R,4R)-7**. Racemic samples were obtained by reduction of ketone **6** under standard Luche conditions (*J. Am. Chem. Soc.* **1978**, 100, 7, 2226–2227; Lanthanides in organic chemistry. 1. Selective 1,2 reductions of conjugated ketones. Jean Louis Luche) : (±)-3-Hydroxy-4,5,5-trimethylcyclopent-1-en-1-yl)methyl pivalate. A solution of 250 mg (1.05 mmol) of ketone **6** in 4 mL of 0.4M $CeCl_3$ in methanol is prepared at 5 ºC. Subsequently, 40 mg (1.05 mmol) of $NaBH_4$ is added in portions over a period of 5 minutes with stirring. The suspension is then allowed to react for a further 5 minutes and poured into water (10 mL), and the mixture was extracted with diethyl ether (2x10 mL). The combined organic layers were washed with brine (10 mL), dried over $MgSO_4$, and concentrated under reduced pressure. The crude residue obtained (248 mg, 98% yield) was directly injected into the GC for the analysis of the diastereomers without further purification.

The best resolution of each diastereomer was obtained under these conditions: the oven temperature was raised at 90 º C and held for one minute, and then at 2°C/min from 90 to 110 °C, and then at 0.3 °C/min from 110 ºC to 150 °C, and then at 30 °C/min from 150 ºC to 220 °C which was finally held for 3 min. The GC-FID chromatograms show. A > 97% of enantiomeric excess could be determined for both products, (3R,4S)-7 and (3R,4R)-7.

Corey–Itsuno reduction products:

(3R,4R)-**7**

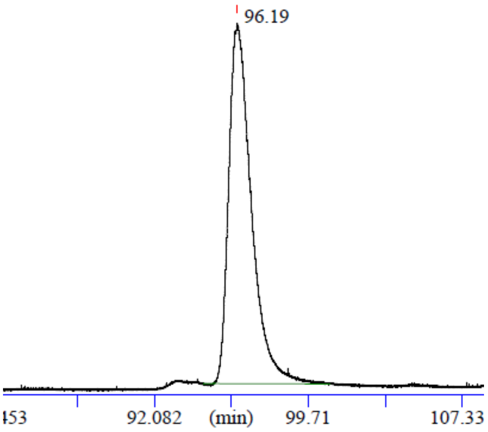

(3R,4S)-**7**

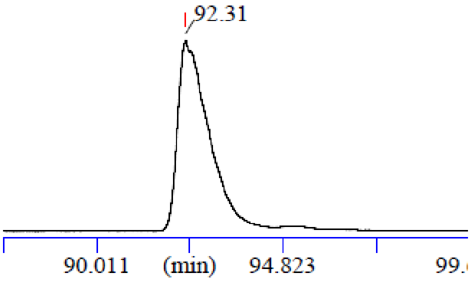

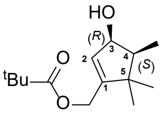

(3*R*,4*S*)-**7**

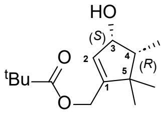

(3*S*,4*R*)-**7**

Luche reduction products:

(3R,4R)-**7** (3S,4S)-**7**

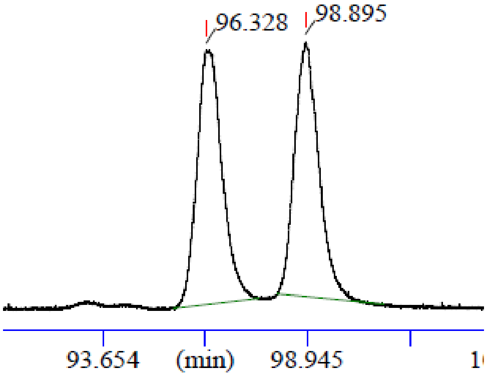

(3R,4S)-**7** (3S,4R)-**7**

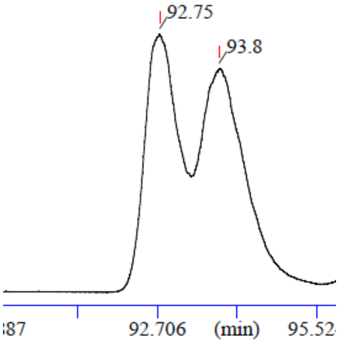

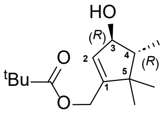

(3*R*,4*R*)-**7**

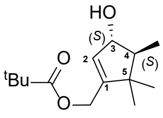

(3*S*,4*S*)-**7**

Crystal data and structure refinement for compound 8.

Table 1 Crystal data and structure refinement for camphanic derivative compound 8

Identification code	b20230204_AA011241DUCu2
Empirical formula	$C_{20}H_{28}O_4$
Formula weight	332.42
Temperature/K	170.00(10)
Crystal system	orthorhombic
Space group	$P2_12_12_1$
a/Å	6.74192(7)
b/Å	10.95860(12)
c/Å	25.7050(3)
α/°	90.0
β/°	90.0
γ/°	90.0
Volume/Å^3	1899.14(4)
Z	4
ρ_{calc}g/cm^3	1.163
μ/mm^{-1}	0.638
F(000)	720.0
Crystal size/mm^3	0.499 × 0.086 × 0.061
Radiation	CuKα (λ = 1.54184)
2Θ range for data collection/°	6.878 to 147.564
Index ranges	$-7 \leq h \leq 8$, $-13 \leq k \leq 13$, $-31 \leq l \leq 31$
Reflections collected	35865
Independent reflections	3817 [R_{int} = 0.1312, R_{sigma} = 0.0477]
Data/restraints/parameters	3817/2/229
Goodness-of-fit on F^2	1.072
Final R indexes [I>=2σ (I)]	R_1 = 0.0482, wR_2 = 0.1277

Final R indexes [all data]	R_1 = 0.0504, wR_2 = 0.1299
Largest diff. peak/hole / e Å^{-3}	0.32/-0.25
Flack parameter	-0.19(16)
Bijvoet Pairs Covarage	99%
Hooft y	-0.19(16)
P3 false	≤10^{-20}

[a] Weighing scheme: $1/[\sigma^2(F_o^2)+(0.0751P)^2 + 0.3034P]$ donde $P = [Max(F_o^2,0)+2F_c^2]/3$.

[b] Expression of secondary extinction type SHELXL: $F_c^* = kF_c[1+0.001F_c^2\lambda^3/sen(2\theta)]^{-1/4}$

Table 2 Fractional Atomic Coordinates (×10^4) and Equivalent Isotropic Displacement Parameters (Å^2×10^3) for b20230204_AA011241DUCu2. U_{eq} is defined as 1/3 of of the trace of the orthogonalised U_{IJ} tensor.

Atom	*x*	*y*	*z*	U(eq)
O1	11660(3)	7782.9(17)	2961.6(8)	41.0(5)
O2	9050(2)	8285.8(15)	3455.7(7)	30.8(4)
O3	4501(3)	9733.8(16)	3825.1(8)	38.3(4)
O4	5602(2)	7793.1(16)	3853.1(7)	33.4(4)
C1	10295(3)	8454(2)	3041.6(10)	30.2(5)
C2	9604(3)	9585(2)	2754.6(9)	28.7(5)
C3	7326(3)	9437(2)	2833.0(9)	28.5(5)
C4	7603(3)	9271(2)	3430.2(9)	27.1(5)
C5	8722(4)	10418(2)	3601.6(10)	32.6(5)
C6	10114(3)	10644(2)	3133.6(10)	32.3(5)
C7	10395(4)	9704(3)	2207.0(10)	39.4(6)
C8	6103(4)	10547(3)	2681.1(11)	39.1(6)
C9	6479(4)	8301(3)	2568.9(10)	38.6(6)
C10	5740(3)	8974(2)	3728.8(9)	27.6(5)
C11	3799(4)	7440(2)	4122.1(10)	33.2(5)
C12	3707(4)	6083(2)	4129.5(9)	30.5(5)
C13	2140(4)	5431(2)	4451.7(10)	35.3(5)
C14	2234(5)	4125(3)	4207.1(13)	48.1(7)
C15	4318(5)	4051(3)	3988.1(12)	46.2(7)
C16	4915(4)	5308(2)	3888.8(10)	36.8(6)
C17	66(4)	5969(4)	4389.5(16)	59.1(9)
C18	2759(5)	5452(4)	5019.4(12)	58.6(9)
C19	1609(6)	3064(3)	4549.7(17)	65.4(10)
C20	5334(7)	3056(4)	3874(2)	76.7(13)

Table 3 Anisotropic Displacement Parameters (Å^2×10^3) for b20230204_AA011241DUCu2. The Anisotropic displacement factor exponent takes the form: $-2\pi^2[h^2a^{*2}U_{11}+2hka^*b^*U_{12}+\ldots]$.

Atom	U_{11}	U_{22}	U_{33}	U_{23}	U_{13}	U_{12}
O1	24.9(8)	40.0(10)	58.2(11)	-1.4(8)	6.7(8)	9.2(7)
O2	21.7(7)	31.6(8)	39.1(8)	4.9(7)	3.9(6)	5.0(7)

O3	29.5(9)	34.3(9)	50.9(10)	2.0(8)	12.5(8)	5.5(8)
O4	24.7(8)	30.5(9)	45.1(9)	5.4(7)	10.7(7)	0.3(7)
C1	16.7(10)	34.4(12)	39.4(12)	-3.6(10)	3.0(9)	1.2(9)
C2	18.3(10)	35.5(12)	32.4(11)	-0.1(9)	1.4(8)	-1.0(9)
C3	17.8(10)	34.8(12)	32.8(11)	1.5(9)	1.6(8)	-1.4(9)
C4	19.2(10)	28.9(11)	33.1(11)	1.6(9)	3.2(8)	3.5(8)
C5	26.3(11)	33.9(12)	37.5(12)	-3.1(10)	2.8(9)	-2.3(10)
C6	21.9(10)	32.8(12)	42.0(13)	-2.1(10)	2.0(9)	-4.7(9)
C7	26.7(12)	54.9(16)	36.5(13)	0.6(11)	7.0(10)	-2.4(12)
C8	22.4(11)	48.0(15)	46.9(14)	12.4(12)	0.5(10)	4.9(11)
C9	24.5(11)	49.2(15)	42.1(13)	-7.8(11)	1.0(10)	-7.9(11)
C10	22.1(10)	29.1(11)	31.5(10)	0.7(9)	2.8(8)	0.2(9)
C11	26.6(11)	34.4(12)	38.6(12)	3.8(10)	11.0(10)	-1.6(10)
C12	26.8(11)	34.5(12)	30.4(11)	2.0(9)	3.0(9)	-3.2(10)
C13	31.7(12)	38.0(13)	36.3(12)	4.0(10)	6.9(10)	-7.0(11)
C14	47.6(16)	44.0(16)	52.7(16)	4.2(13)	-1.6(13)	-10.3(13)
C15	50.8(16)	40.0(14)	47.8(15)	-1.6(12)	6.5(13)	0.4(13)
C16	32.0(12)	38.6(14)	39.8(13)	1.8(10)	8.1(10)	2.3(10)
C17	32.2(15)	70(2)	75(2)	21.4(18)	9.0(15)	-3.9(15)
C18	62(2)	79(2)	35.4(15)	9.7(15)	2.8(14)	-20.5(19)
C19	64(2)	48.3(19)	84(2)	15.5(17)	11.2(19)	-14.0(17)
C20	83(3)	38.8(17)	108(3)	2.4(19)	34(3)	11.6(19)

Table 4 Bond Lengths for b20230204_AA011241DUCu2.

Atom	Atom	Length/Å	Atom	Atom	Length/Å
O1	C1	1.196(3)	C4	C5	1.532(3)
O2	C1	1.368(3)	C4	C10	1.507(3)
O2	C4	1.456(3)	C5	C6	1.546(3)
O3	C10	1.205(3)	C11	C12	1.489(3)
O4	C10	1.337(3)	C12	C13	1.521(3)
O4	C11	1.451(3)	C12	C16	1.329(4)
C1	C2	1.516(3)	C13	C14	1.564(4)
C2	C3	1.558(3)	C13	C17	1.526(4)
C2	C6	1.554(3)	C13	C18	1.518(4)
C2	C7	1.511(3)	C14	C15	1.516(5)
C3	C4	1.557(3)	C14	C19	1.518(4)
C3	C8	1.521(4)	C15	C16	1.457(4)
C3	C9	1.529(3)	C15	C20	1.321(5)

Table 5 Bond Angles for b20230204_AA011241DUCu2.

Atom	Atom	Atom	Angle/°	Atom	Atom	Atom	Angle/°
C1	O2	C4	106.04(17)	C4	C5	C6	101.91(19)

C10	O4	C11	115.46(18)	C5	C6	C2	103.55(18)
O1	C1	O2	121.6(2)	O3	C10	O4	124.9(2)
O1	C1	C2	131.0(2)	O3	C10	C4	122.2(2)
O2	C1	C2	107.47(18)	O4	C10	C4	112.83(19)
C1	C2	C3	98.90(18)	O4	C11	C12	107.9(2)
C1	C2	C6	103.73(19)	C11	C12	C13	120.4(2)
C6	C2	C3	102.43(19)	C16	C12	C11	127.4(2)
C7	C2	C1	114.5(2)	C16	C12	C13	112.2(2)
C7	C2	C3	118.55(19)	C12	C13	C14	100.5(2)
C7	C2	C6	116.2(2)	C12	C13	C17	113.4(2)
C4	C3	C2	91.23(17)	C17	C13	C14	110.4(3)
C8	C3	C2	114.7(2)	C18	C13	C12	109.0(2)
C8	C3	C4	114.3(2)	C18	C13	C14	112.9(3)
C8	C3	C9	109.6(2)	C18	C13	C17	110.3(3)
C9	C3	C2	113.3(2)	C15	C14	C13	103.6(2)
C9	C3	C4	112.8(2)	C15	C14	C19	115.6(3)
O2	C4	C3	102.20(18)	C19	C14	C13	117.1(3)
O2	C4	C5	105.40(18)	C16	C15	C14	105.7(2)
O2	C4	C10	112.08(18)	C20	C15	C14	127.4(3)
C5	C4	C3	104.29(18)	C20	C15	C16	126.7(3)
C10	C4	C3	115.29(18)	C12	C16	C15	110.7(2)
C10	C4	C5	116.2(2)				

Table 6 Torsion Angles for b20230204_AA011241DUCu2.

A	B	C	D	Angle/°	A	B	C	D	Angle/°
O1	C1	C2	C3	145.6(3)	C7	C2	C3	C8	-66.6(3)
O1	C1	C2	C6	-109.2(3)	C7	C2	C3	C9	60.2(3)
O1	C1	C2	C7	18.5(4)	C7	C2	C6	C5	167.0(2)
O2	C1	C2	C3	-35.6(2)	C8	C3	C4	O2	-171.37(19)
O2	C1	C2	C6	69.6(2)	C8	C3	C4	C5	-61.8(2)
O2	C1	C2	C7	-162.7(2)	C8	C3	C4	C10	66.8(3)
O2	C4	C5	C6	70.9(2)	C9	C3	C4	O2	62.6(2)
O2	C4	C10	O3	168.0(2)	C9	C3	C4	C5	172.17(19)
O2	C4	C10	O4	-14.3(3)	C9	C3	C4	C10	-59.3(3)
O4	C11	C12	C13	171.2(2)	C10	O4	C11	C12	168.4(2)
O4	C11	C12	C16	-6.8(4)	C10	C4	C5	C6	-164.36(19)
C1	O2	C4	C3	35.2(2)	C11	O4	C10	O3	0.2(3)
C1	O2	C4	C5	-73.6(2)	C11	O4	C10	C4	-177.49(19)
C1	O2	C4	C10	159.21(19)	C11	C12	C13	C14	163.8(2)
C1	C2	C3	C4	51.45(19)	C11	C12	C13	C17	45.9(4)
C1	C2	C3	C8	169.0(2)	C11	C12	C13	C18	-77.3(3)

C1	C2	C3	C9	-64.1(2)	C11	C12	C16	C15	-179.4(3)
C1	C2	C6	C5	-66.4(2)	C12	C13	C14	C15	25.2(3)
C2	C3	C4	O2	-53.44(19)	C12	C13	C14	C19	153.8(3)
C2	C3	C4	C5	56.2(2)	C13	C12	C16	C15	2.5(3)
C2	C3	C4	C10	-175.3(2)	C13	C14	C15	C16	-25.2(3)
C3	C2	C6	C5	36.1(2)	C13	C14	C15	C20	159.3(4)
C3	C4	C5	C6	-36.3(2)	C14	C15	C16	C12	14.9(3)
C3	C4	C10	O3	-75.7(3)	C16	C12	C13	C14	-17.9(3)
C3	C4	C10	O4	102.1(2)	C16	C12	C13	C17	-135.8(3)
C4	O2	C1	O1	179.4(2)	C16	C12	C13	C18	100.9(3)
C4	O2	C1	C2	0.5(2)	C17	C13	C14	C15	145.3(3)
C4	C5	C6	C2	-0.1(2)	C17	C13	C14	C19	-86.1(3)
C5	C4	C10	O3	46.8(3)	C18	C13	C14	C15	-90.7(3)
C5	C4	C10	O4	-135.5(2)	C18	C13	C14	C19	37.9(4)
C6	C2	C3	C4	-54.8(2)	C19	C14	C15	C16	-154.7(3)
C6	C2	C3	C8	62.8(3)	C19	C14	C15	C20	29.8(6)
C6	C2	C3	C9	-170.4(2)	C20	C15	C16	C12	-169.5(4)
C7	C2	C3	C4	175.8(2)					

Table 7 Hydrogen Atom Coordinates (Å×10^4) and Isotropic Displacement Parameters (Å^2×10^3) for b20230204_AA011241DUCu2.

Atom	*x*	*y*	*z*	U(eq)
H5A	9482.78	10272.94	3925.11	39
H5B	7806	11113.28	3655.45	39
H6A	11522.99	10609.29	3241.23	39
H6B	9847.02	11446.49	2971.46	39
H7A	11845.2	9761.76	2217.07	59
H7B	9848.2	10440.65	2045.33	59
H7C	10005.43	8986.82	2003.49	59
H8A	4737.01	10442.17	2803.81	59
H8B	6105.16	10634.54	2301.7	59
H8C	6677.94	11278.58	2839.89	59
H9A	7243.65	7582.43	2675.36	58
H9B	6559.85	8395.86	2190.41	58
H9C	5089.19	8195.36	2671.57	58
H11A	2626.65	7776.03	3939.52	40
H11B	3809.71	7762.24	4481.94	40
H14	1311.85	4129.46	3902.43	58
H16	6015.48	5540.73	3680.64	44
H17A	9.48	6766.87	4561.29	89
H17B	-908.87	5420.58	4548.55	89
H17C	-235.04	6066.23	4018.83	89
H18A	4022.48	5020.22	5059.83	88
H18B	1740.65	5050.9	5230.87	88

H18C	2914.22	6299.82	5134.1	88
H19A	250.75	3196.44	4672.95	98
H19B	2506.88	3005.81	4848.58	98
H19C	1667.35	2305.01	4348.72	98
H20A	6540(40)	3090(50)	3714(18)	92
H20B	4830(70)	2310(20)	3950(20)	92

Experimental

Single crystals of $C_{20}H_{28}O_4$ **[b20230204_AA011241DUCu2]**. A suitable crystal was selected and **mounted** on a **SuperNova, Dual, Cu at home/near, HyPix** diffractometer. The crystal was kept at 170.00(10) K during data collection. Using Olex2 [1], the structure was solved with the ShelXT [2] structure solution program using Intrinsic Phasing and refined with the ShelXL [3] refinement package using Least Squares minimisation.

1. Dolomanov, O.V., Bourhis, L.J., Gildea, R.J, Howard, J.A.K. & Puschmann, H. (2009), J. Appl. Cryst. 42, 339-341.
2. Sheldrick, G.M. (2015). Acta Cryst. A71, 3-8.
3. Sheldrick, G.M. (2015). Acta Cryst. C71, 3-8.

Crystal structure determination of [b20230204_AA011241DUCu2]

Crystal Data for $C_{20}H_{28}O_4$ (M =332.42 g/mol): orthorhombic, space group $P2_12_12_1$ (no. 19), a = 6.74192(7) Å, b = 10.95860(12) Å, c = 25.7050(3) Å, V = 1899.14(4) $Å^3$, Z = 4, T = 170.00(10) K, μ(CuKα) = 0.638 mm^{-1}, $Dcalc$ = 1.163 g/cm^3, 35865 reflections measured (6.878° ≤ 2Θ ≤ 147.564°), 3817 unique (R_{int} = 0.1312, R_{sigma} = 0.0477) which were used in all calculations. The final R_1 was 0.0482 (I > 2σ(I)) and wR_2 was 0.1299 (all data).

Refinement model description

Number of restraints - 2, number of constraints - unknown.

Details:

```
1. Fixed Uiso
 At 1.2 times of:
  All C(H) groups, All C(H,H) groups
 At 1.5 times of:
  All C(H,H,H) groups
2. Restrained distances
 C20-H20B = C20-H20A
 0.9 with sigma of 0.01
3.a Ternary CH refined with riding coordinates:
 C14(H14)
3.b Secondary CH2 refined with riding coordinates:
 C5(H5A,H5B), C6(H6A,H6B), C11(H11A,H11B)
3.c Aromatic/amide H refined with riding coordinates:
 C16(H16)
3.d Idealised Me refined as rotating group:
 C7(H7A,H7B,H7C), C8(H8A,H8B,H8C), C9(H9A,H9B,H9C), C17(H17A,H17B,H17C),
 C18(H18A,H18B,H18C), C19(H19A,H19B,H19C)
```

This report has been created with Olex2, compiled on 2018.05.29 svn.r3508 for OlexSys. Please let us know if there are any errors or if you would like to have additional features.

Analysis of the absolute structure using likelihood methods (Hooft, Straver & Spek, 2008 [4]) was performed using PLATON (Spek, 2010[3]). The Friedel pair coverage of the experiment is almost complete (99%). The results indicated that the absolute structure had been correctly assigned. The method calculated that the probability that the structure is inverted is smaller than 10^{-20}. The absolute structure parameter y (Hooft, Straver & Spek, 2008[4]) was calculated using PLATON (Spek, 2010[3]). The resulting value was y=-0.19(12), which together with Flack parameter value, indicate that the absolute structure has probably been determined correctly.

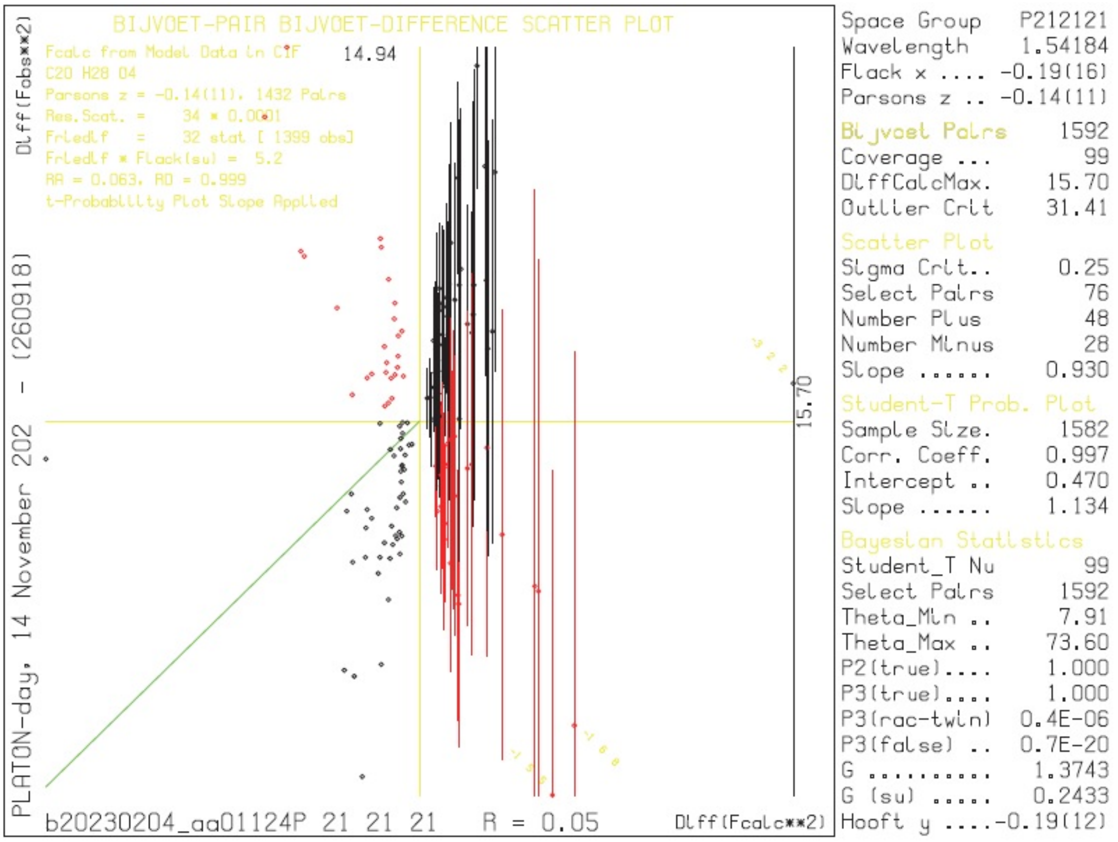

Figure 1. Analysis of the absolute structure by Bayesian statistics using the Platon program.

[1] H. D. Flack & G. Bernardinelli, Acta Cryst. 1999, A55, 908-915; H. D. Flack & G. Bernardinelli, J. Appl. Cryst. 2000, 33, 1143-1148.
[2] R. W. W. Hooft, L. H. Straver & A. L. Spek, J. Appl. Cryst. 2008, 41, 96-103
[3] A. L. Spek (2010) PLATON, A Multipurpose Crystallographic Tool, Utrecht University, Utrecht, The Netherlands; A. L. Spek, J. Appl. Cryst. 2003, 36, 7-13.
[4] A. L. Thompson & D. J. Watkin, Tetrahedron: Asymmetry 2009, 20, 712–717
